# Condensed Supramolecular Helices: The Twisted Sisters of DNA

**DOI:** 10.1002/anie.202113279

**Published:** 2021-12-03

**Authors:** Guanqun Du, Domagoj Belić, Alessandra Del Giudice, Viveka Alfredsson, Anna M. Carnerup, Kaizheng Zhu, Bo Nyström, Yilin Wang, Luciano Galantini, Karin Schillén

**Affiliations:** ^1^ Division of Physical Chemistry Department of Chemistry Lund University P.O. Box 124 22100 Lund Sweden; ^2^ Department of Chemistry Sapienza University of Rome P.O. Box 34-Roma 62, Piazzale A. Moro 5 00185 Roma Italy; ^3^ Department of Chemistry University of Oslo P.O. Box 1033, Blindern 0315 Oslo Norway; ^4^ Key Laboratory of Colloid and Interface Science Beijing National Laboratory for Molecular Sciences Institute of Chemistry Chinese Academy of Sciences Beijing 100190 China; ^5^ Department of Physics Josip Juraj Strossmayer University of Osijek 31000 Osijek Croatia

**Keywords:** chirality, condensation, DNA-like helix superstructures, hexagonally packed helices, polyelectrolyte–bile salt systems

## Abstract

Condensation of DNA helices into hexagonally packed bundles and toroids represents an intriguing example of functional organization of biological macromolecules at the nanoscale. The condensation models are based on the unique polyelectrolyte features of DNA, however here we could reproduce a DNA‐like condensation with supramolecular helices of small chiral molecules, thereby demonstrating that it is a more general phenomenon. We show that the bile salt sodium deoxycholate can form supramolecular helices upon interaction with oppositely charged polyelectrolytes of homopolymer or block copolymers. At higher order, a controlled hexagonal packing of the helices into DNA‐like bundles and toroids could be accomplished. The results disclose unknown similarities between covalent and supramolecular non‐covalent helical polyelectrolytes, which inspire visionary ideas of constructing supramolecular versions of biological macromolecules. As drug nanocarriers the polymer–bile salt superstructures would get advantage of a complex chirality at molecular and supramolecular levels, whose effect on the nanocarrier assisted drug efficiency is a still unexplored fascinating issue.

## Graphical abstract

Chiral bile salt biosurfactant with low molecular weight can self‐assemble into supramolecular helices by interaction with oppositely charged polymers. A controlled condensation of the supramolecular helices into DNA‐like hexagonally packed bundles and toroids can be achieved when using block copolymers of opposite charge and increasing the bile salt content.

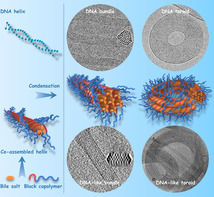

## Introduction

Folding and condensation of chiral macromolecules on the nano‐ and micro‐scale play fundamental roles in living organisms. The helix of DNA, perhaps the most famous example of chirality in molecular biology, condensates into higher‐order supramolecular structures including bundles, toroids, and chromatin.^[1]^ Condensation allows it to fulfil fundamental biological functions and to fit into compartments like cells and virus capsids.^[2]^ It also enables the DNA molecule to be loaded efficiently into gene delivery carriers to provide advanced applications in nanotechnology.^[3]^

Small chiral molecules can associate via non‐covalent interactions into aggregates with a supramolecular chirality,^[4]^ thereby providing a versatile alternative to reproduce the features of chiral macromolecules. This can be exploited in applications such as development of functional materials for chiro‐optical switches, assisted synthesis of chiral nanoparticles^[5]^ and chiral recognition, catalysis and luminescence.^[6]^

In a hierarchical assembly, small chiral molecules can easily form supramolecular helices at low association level, thereby mimicking the local helical structures of macromolecules. By contrast, it is particularly challenging to reproduce the complex structures of macromolecules at larger scale, e.g., at tertiary and quaternary levels, where a covalent binding between monomers is generally necessary to guarantee the mechanical features and the stability of the folding and association of the macromolecule chains. The formation of such high‐order complex structures is relevant to gain deep understanding of supramolecular self‐assembly fundaments. Their controlled formation can open new, still unexplored avenues, of applications of self‐assembly materials.

**Scheme 1:**
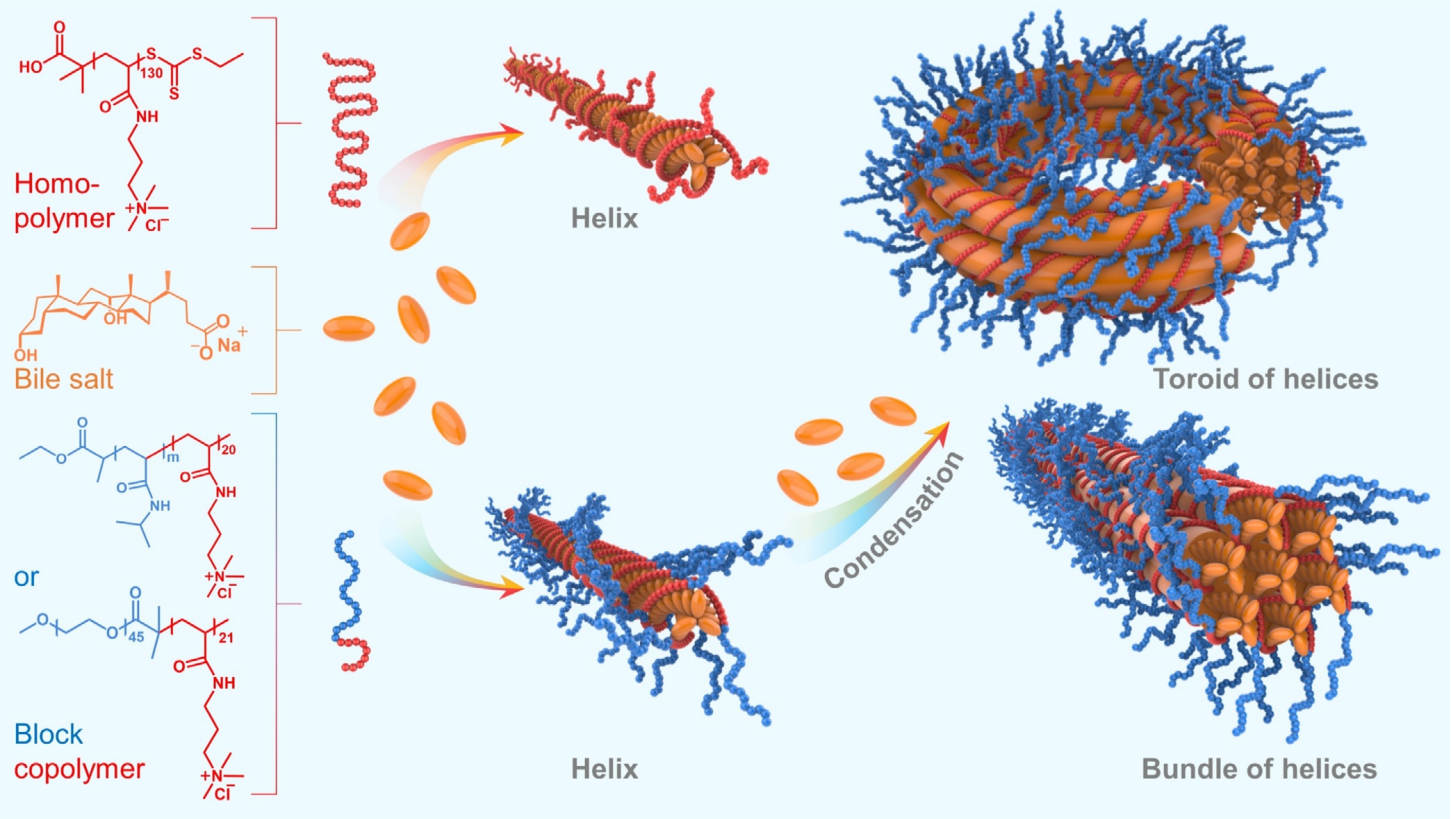
Illustration of supramolecular helix formation and condensation. Chemical structures of PAMPTMA(+)_130_ homopolymer (left panel, top), bile salt NaDC (left panel, middle), and PNIPAM_*m*_‐*b*‐PAMPTMA(+)_20_ (*m=*65 or 48) and MPEG_45_‐*b*‐PAMPTMA(+)_21_ block copolymers (left panel, bottom). NaDC helix formation induced by interaction with homopolymer (center panel, top) or block copolymers (center panel, bottom). Condensation of block copolymer–NaDC helices into toroid (right panel, top) and bundle (right panel, bottom). Color code: red (PAMPTMA(+)), blue (PNIPAM or MPEG), orange (NaDC).

In this work, we were able to control the hierarchical self‐assembly of sodium deoxycholate (NaDC), a chiral steroid and anionic biosurfactant belonging to the bile salt (BS) family, up to DNA condensed‐like structures.[^1a^, ^1f^] The self‐assembly was induced by the interaction with oppositely charged homopolymer or block copolymers and could be directed by tuning the charge ratio of the polymer–BS mixture, as well as the chemical composition of the polymers. Single supramolecular helices were mainly formed at low negative charge fractions, and they were clearly isolated when using the homopolymer. With block copolymers, an assembly of the helices into hexagonally organized bundles and sometimes toroidal‐like structures, like those formed by DNA condensation, was induced by increasing the bile salt fraction in the mixture (Scheme 1).

## Results and Discussion

Mixtures of the NaDC and poly((3‐acrylamidopropyl)trimethylammonium chloride) (PAMPTMA(+)_130_) homopolymer or diblock copolymers composed of one cationic PAMPTMA(+) block and a nonionic block of either poly(*N*‐isopropylacrylamide) (PNIPAM) (denoted PNIPAM_*m*_‐*b*‐PAMPTMA(+)_20_, *m=*65 or 48)^[7]^ or methoxy‐poly(ethylene glycol) (MPEG) (denoted MPEG_45_‐*b*‐PAMPTMA(+)_21_)^[8]^ were characterized as a function of the charge ratio (*CR*). *CR* is defined as the number of moles of negative charge (here NaDC) divided by the number of moles of positive charge (=polymerization degree of PAMPTMA(+) block × number of moles of block copolymer). A mixed solution of NaDC and PNIPAM_71_ homopolymer^[7]^ at a molar ratio, *MR*=0.3 (*MR*=the number of moles of NaDC/[71 × number of moles of PNIPAM_71_]), was also investigated. Detailed experiments can be found in Supporting Information (SI).

**Figure 1:**
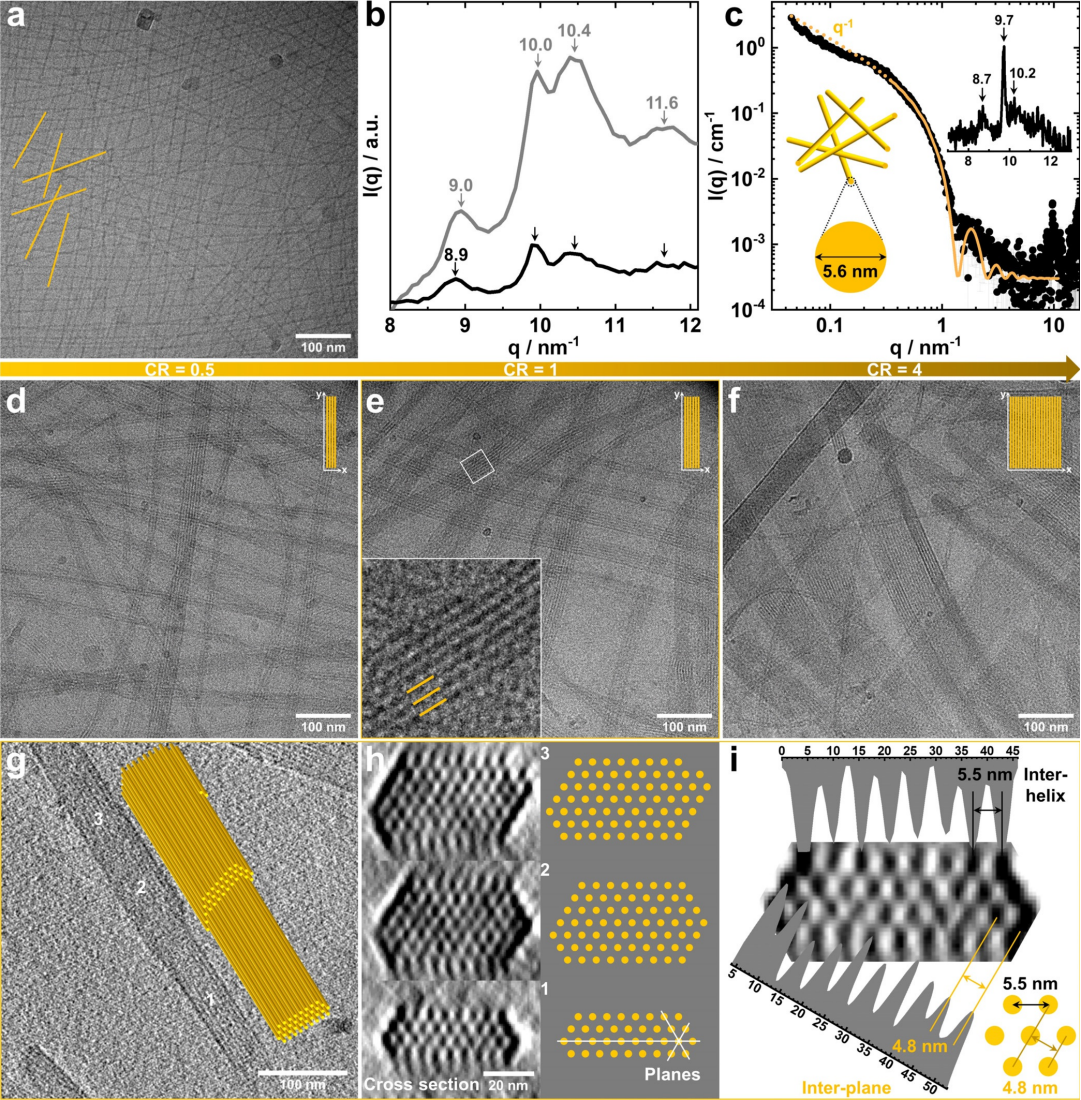
a) Cryo‐TEM image at *CR*=0.5 and b) WAXS curves of the concentrated phase at *CR*=0.5 (black) and the precipitate at *CR*=1 (gray) of PAMPTMA(+)_130_–NaDC mixtures. The *q* (the magnitude of the scattering vector) values of the WAXS peaks are indicated in (b). c) Experimental SAXS curve (scattering intensity I(*q*) versus *q* (black symbols) of the same sample as in (a) and best‐fitting curve (orange line) for a model of rod‐like particles with a cross‐section diameter of 5.6 nm (inset: WAXS region with the peaks indicated). Cryo‐TEM images of PNIPAM_65_‐*b*‐PAMPTMA(+)_20_–NaDC mixtures at *CR* of d) 0.5, e) 1 (inset: enlargement displaying the bundle interior in the marked region), and f) 4. g) Top view cryo‐ET reconstruction and corresponding 3D sketched model of bundles of the supramolecular helices in the PNIPAM_65_‐*b*‐PAMPTMA(+)_20_–NaDC mixed complexes at *CR*=1. h) Cross sections and corresponding sketches reconstructed in different positions of (g) marked with 1, 2, and 3. i) Gray‐scale analysis of the cross section and sketch of the hexagonal lattice with inter‐helix (5.5 nm) and inter‐plane (4.8 nm) distances.

Cryogenic transmission electron microscopy (cryo‐TEM) images of aqueous mixtures of PAMPTMA(+)_130_ (0.5 wt %) and NaDC (12.0 mM) at *CR*=0.5 revealed micrometer‐long nanowires (Figure 1 a). It is well established that NaDC forms gels at pH of about 7,^[9]^ from which dry fibers can be drawn, consisting of NaDC molecules in a helical arrangement as reflected in a very typical X‐ray pattern.[^9a^, ^10^] A wide‐angle X‐ray scattering (WAXS) experiment was performed on a concentrated PAMPTMA(+)_130_–NaDC mixture at *CR*=0.5 (Figure 1 b) revealing peaks that matched the layer lines of the dry fiber X‐ray pattern ascribed to the typical supramolecular helical structure of NaDC (see description in SI).[^9a^, ^10b^] A similar pattern was obtained for a NaDC gel formed at neutral pH (Figure S1a, inset) and a dilute PAMPTMA(+)_130_–NaDC mixed solution (Figure 1 c, inset). From this, we concluded that the PAMPTMA(+)_130_–NaDC nanowires were composed of helices of deoxycholate anions (DC^−^) with PAMPTMA(+)_130_ polyions associated through electrostatic interaction. The helices had a structure very similar to those found in NaDC gels and dry fibers. We recorded a SAXS pattern of oriented rod‐like particles for the mixture at *CR*=0.5 (Figure S2), which is consistent with the cryo‐TEM result in Figure 1 a. Based on a model of homogeneous cylinders a cross‐section diameter of 5.6 nm was estimated from the best‐fit averaged SAXS curve (Figure 1 c), in agreement with the diameter estimated from the cryo‐TEM image (Figure S3). A slightly larger maximum cross‐section distance (≈7 nm) was inferred by the Indirect Fourier Transform (IFT) pair correlation function (Figure S1b) suggesting a non‐homogeneous cross section of the helices. Slightly smaller cross‐section diameter (4.4 nm, Figure S1a) and maximum cross‐section distance (≈5 nm, Figure S1b) were inferred from the model‐based fit and IFT for the pH‐induced NaDC gel, reasonably due to the contribution of the shell of PAMPTMA(+)_130_ chains associated to the helices in the polyelectrolyte–NaDC mixture. A phase separation into a white precipitate in equilibrium with a dilute phase occurred in the mixture at *CR*=1 and the WAXS pattern revealed the precipitate to consist of helices (Figure 1 b). More information on the phase behavior of the PAMPTMA(+)_130_–NaDC mixtures at different *CR*s is reported in Figure S4.

Linking a nonionic polymer block to the polyelectrolyte chain promoted the helices to assemble into bundles as shown in cryo‐TEM images of mixtures of PNIPAM_*m*_‐*b*‐PAMPTMA(+)_20_ diblock copolymers (0.1 wt %) and NaDC (0.9–8.3 mM) (Figures 1 d–f and S5–S12). The bundles consisted of orderly packed helices with a regular spacing at all *CR*s, which co‐existed with single helices in the mixtures when *CR*≤0.5 (Figures S5a and S9). The single helices had a similar appearance as those found in PAMPTMA(+)_130_–NaDC system (Figure 1 a). Irrespectively of the PNIPAM block length of the investigated PNIPAM_*m*_‐*b*‐PAMPTMA(+)_20_ copolymers, the width of the bundles became wider with increasing *CR*, thus being tuneable through optimization of the mixing ratio (Figures 1 d–f, S5, and Table S1). These results show that the phase separation observed in the PAMPTMA(+)_130_–NaDC system at *CR*=1 and 2 (see Figure S4) can be avoided, i.e., it is possible to control the colloidal stability of the system, by using cationic diblock copolymers with a nonionic water‐soluble block. Indeed, the co‐assembled structures were stable in solution at room temperature for a surprisingly long time (at least for several months). At elevated temperatures above the phase transition of PNIPAM, which is thermoresponsive, the colloidal stability decreases as found for a similar block copolymer–NaDC system.^[11]^

Analysis of the cross section of the bundle obtained from the cryogenic electron tomography (cryo‐ET) 3D reconstruction of a representative PNIPAM_65_‐*b*‐PAMPTMA(+)_20_–NaDC mixture at *CR*=1 (Figure S13 and Movie S1) revealed that the supramolecular helices were packed in a hexagonal lattice (Figures 1 h, S14b, and Movies S2, S3). An inter‐helix distance of 5.5 nm and an inter‐plane distance of 4.8 nm was estimated from the gray value analysis (Figure 1 i). The distances were in good agreement with the periodic spacing in the bundles generally measured from the cryo‐TEM 2D images (Figures S6–S8 and S10–S12) and also consistent with those of the hexagonal liquid‐crystalline phase at high NaDC concentration^[12]^ (more details about the cryo‐ET analysis can be found in SI).

**Figure 2:**
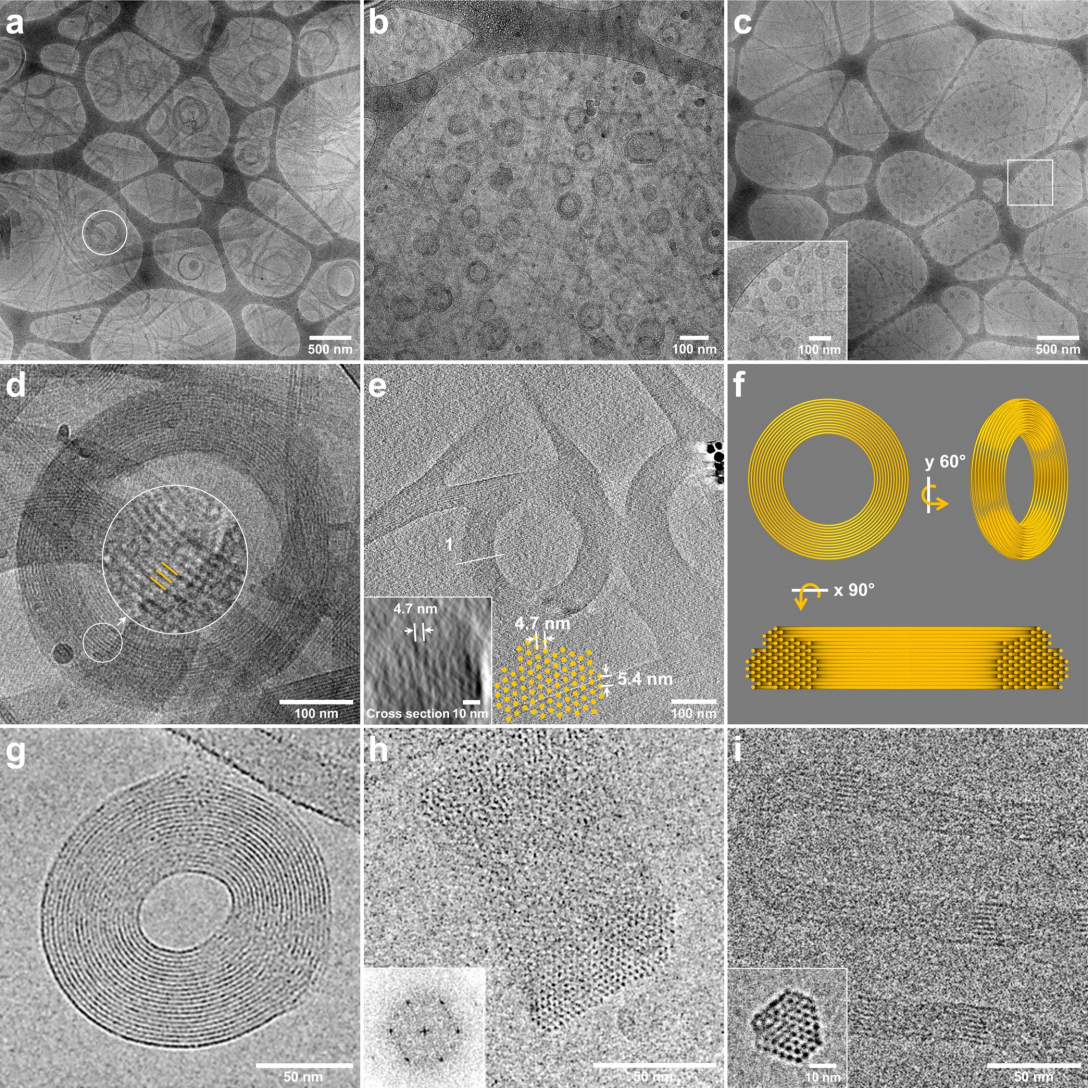
Cryo‐TEM overview showing the toroidal structures of a) MPEG_45_‐*b*‐PAMPTMA(+)_21_–NaDC mixture at *CR*=1, b) PNIPAM_65_‐*b*‐PAMPTMA(+)_20_–NaDC mixture at *CR*=0.5, and c) PNIPAM_48_‐*b*‐PAMPTMA(+)_20_–NaDC mixture at *CR*=1, with a zoomed‐in area of (c) in the inset. d) Zoom in of the toroid marked in (a) together with a further zoomed‐in area highlighting the internal structure. e) Cryo‐ET reconstruction of a toroid of the MPEG_45_‐*b*‐PAMPTMA(+)_21_–NaDC mixture at *CR*=1 with the reconstructed cross section and inter‐plane distance reported in the inset and reproduced in the sketch. f) 3D model sketches of the toroid in (e) displaying different orientations and a cross section with hexagonal lattice achieved by a 90° rotation of the model. Cryo‐electron micrographs of λ DNA toroids g) from top view and h) oriented ≈90° with respect to the image plane (inset: Fourier transform of hexagonally ordered DNA lattice; adapted with the permission from ref. [1f], Copyright (2001) National Academy of Sciences, U.S.A.). i) Cryo‐TEM image (top view and cross section in the inset) of polyelectrolyte complex micelles with parallel ordering of DNA helices composed of double‐stranded 22‐bp DNA and cationic block copolymer (Adapted with the permission from ref. [1a], Copyright © 2018 American Chemical Society).

The cryo‐TEM experiments performed on MPEG_45_‐*b*‐PAMPTMA(+)_21_–NaDC mixtures at *CR*=0.5 (Figure S15) and *CR*=1 (Figure 2 a,d) showed the same type of bundles as observed for the PNIPAM_*m*_‐*b*‐PAMPTMA(+)_20_–NaDC systems. Single helices co‐existed with such bundles at *CR*=0.5 (Figure S15). The periodic distance measured in the bundles was similar to the distance obtained for the PNIPAM_*m*_‐*b*‐PAMPTMA(+)_20_–NaDC systems (Figure S16d,e).

Toroidal structures were found to co‐exist with bundles in all the investigated block copolymer–NaDC systems. The toroid sizes, in terms of average outer diameter *D* and thickness *T* of the toroidal wall, are summarized in Table S1. The toroids of the PNIPAM_*m*_‐*b*‐PAMPTMA(+)_20_–NaDC systems had a slightly larger diameter (*D*=81±15 nm) in the case of the copolymer with the longer PNIPAM block (*m=*65) compared to the copolymer with the shorter one (*m=*48) (*D*=55±10 nm), while the thickness was roughly the same in both cases (15–17 nm) (Figures S10, S7, and Table S1). The toroids of the MPEG_45_‐*b*‐PAMPTMA(+)_21_–NaDC system were in general much larger (*D*=395±81 nm and *T=*75±25 nm) than those of the PNIPAM_*m*_‐*b*‐PAMPTMA(+)_20_–NaDC systems (Figure S16a–c and Table S1), and displayed a better resolved internal structure (Figure 2 d). Both the 2D cryo‐TEM experiments (Figure S17c) and the cryo‐ET reconstructions indicate that the toroids were built up by concentrically and hexagonally packed helices with an inter‐plane distance of 4.7 nm and a calculated inter‐helix distance of 5.4 nm (Figures 2 e (inset), S17e, and Movies S4–S6). This is in good correlation with the distances obtained for the bundle of the same sample (Figure S17f) and in the PNIPAM_*m*_‐*b*‐PAMPTMA(+)_20_–NaDC systems (Figure 1) (more cryo‐ET analysis is available in SI).

Similarly to the PAMPTMA(+)_130_–NaDC system, all block copolymer–NaDC mixtures showed a WAXS pattern (Figure S18) with peaks corresponding to the main layer lines of the dried NaDC fiber,[^9a^, ^10b^] and therefore confirming that bundles and toroids are built up by hexagonally close‐packed BS helices. The hexagonal packing was also evidenced by a main peak in the SAXS pattern (Figures S18 and S19), which was invariant irrespective of copolymer used (see details in SI).

Strikingly, the bundles and toroids of the block copolymer–NaDC mixtures were structurally very similar to those formed by hexagonally close‐packed DNA helices under specific conditions (Figure 2 g–i). DNA toroids, which have attracted considerable attention, can form in vivo in sperm cells and icosahedral viruses,[^2^, ^13^] as well as in vitro, e.g., in mixtures with condensing agents like polyamine spermidine, multi‐ and mono‐valent cations, dendrimers or block copolymers.[^1a^, ^1e^, ^1f^, ^1g^, ^1h^, ^14^] The inter‐helical distance in the hexagonal arrays of DNA helices is 2.8 nm,[^1f^, ^1g^] roughly half of the distance observed in our systems. Generally, DNA toroids have an outer diameter of about 100 nm and a thickness around 35 nm, although larger outer diameters were previously observed, for instance, in the presence of mixtures of counterions (200 nm) or with the aid of bacteriophages (300 nm).^[1g]^ The toroid size of the copolymer–NaDC systems studied here was of the same order of magnitude but spanned larger ranges for the average diameter (55–400 nm) and the thickness (15–80 nm).

Under condensing conditions, DNA toroids are formed through the balance between effective attractive interactions (favoring compaction), and contributions related to the bending rigidity or curvature stress in addition to excluded volume interactions (opposing compaction).[^2a^, ^14a^] The formation is promoted by different mechanisms involving electrostatics, hydration forces, confinement, and crowding.[^1g^, ^1h^, ^15^] The principles for DNA condensation cannot be strictly applied in our system that is of a supramolecular nature. However, we could assume a well‐separated simplified two‐step process comprising the formation of the NaDC helices (first step) and their condensation into toroids and bundles (second step). Under this assumption, a mechanism based on the principles of DNA condensation could be plausible for the second step. Even though our results clearly demonstrate that the interactions between polymer and NaDC play a vital role in the formation of superstructures, the data collected so far is not sufficient to discriminate between possible mechanisms of formation or rationalize the size distribution of the structures. Rationalizing from a qualitative interaction model of mixtures of polyelectrolytes and oppositely charged surfactants, the helices are expected to form as a consequence of the self‐assembly of the DC^−^ anions onto the polyelectrolyte chain due to the attractive electrostatic interaction and the entropy gain related to the release of counterions, as well as surfactant hydrophobic interactions.^[16]^

The microscopy and X‐ray techniques demonstrated that the lattice of the packed helices was the same irrespective of the length and the chemical composition of the nonionic polymer block (either the amphiphilic PNIPAM or the hydrophilic MPEG). This suggests that the core of the bundles and toroids was mostly comprised of helices of DC^−^ anions, while the charged blocks of the copolymers acted as large counterions at the surface and/or in a thin outermost shell of the core with the nonionic blocks protruding into the surrounding solution (Scheme 1). We foresee that also small counterions, like sodium ions or protons, could neutralize and electrostatically stabilize the deep interior of these structures. The protonation of DC^−^ anions is particularly likely, as it is well known that NaDC is very prone to form pH‐induced gels of supramolecular helices at relatively high pH (about 7, depending on the concentration).^[9a]^

To verify the proposed structural model as depicted in Scheme 1, liquid ^1^H NMR was employed (Figure S20). When internal dynamic motion is restricted, the nuclear spin–spin relaxation (or T2 relaxation) is decreased, which leads to a broadening of the line width.^[17]^ In agreement with the model, the ^1^H NMR spectrum on the PNIPAM_48_‐*b*‐PAMPTMA(+)_20_–NaDC mixed solution at *CR*=1 (Figure S20c) revealed that the protons in the PAMPTMA(+) block bound to the complex surface showed a broadening of the signals due to a restricted motion. The effect was more prominent for the protons of the DC^−^ anions, for which the signal almost vanished due to tight packing of the BS molecules in the helices. By contrast, the broadening was much less relevant for the protons of the PNIPAM block, suggesting that this block was mainly free to move in the solution except for a short segment close to the surface of the complex.

**Figure 3:**
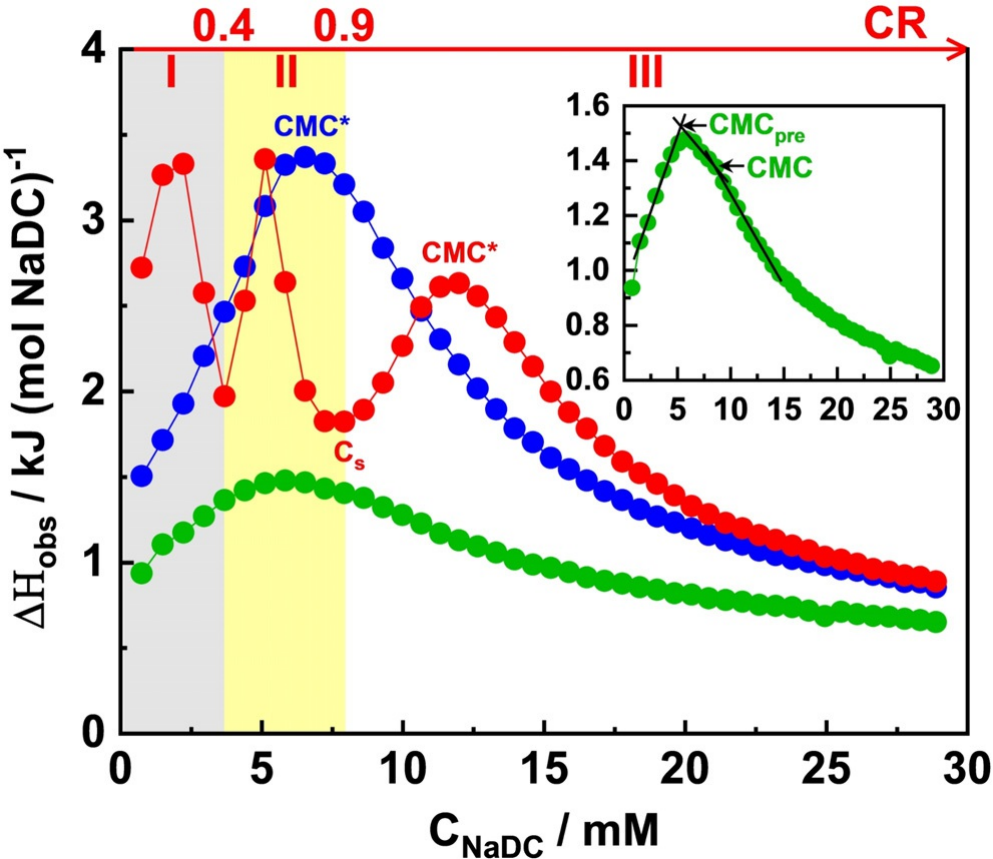
ITC curves for the titration of 200 mM NaDC solution into water (green), PNIPAM_71_ solution (blue), and PNIPAM_65_‐*b*‐PAMPTMA(+)_20_ solution (red). The polymer concentration was 0.5 wt %. The inset displays the ITC curve of the NaDC titrated into water in which the CMC_pre_ and CMC are marked.

ITC allowed us to gain relevant insights on thermodynamics and mechanism of formation of the supramolecular block copolymer–BS mixed complexes.^[18]^ Titration of micellar solution of NaDC (200 mM) into water (dilution) and into PNIPAM_71_ homopolymer or PNIPAM_*m*_‐*b*‐PAMPTMA(+)_20_ block copolymer solutions (0.5 wt %) was carried out in order to discriminate the contributions of the different interactions involved in the complex formation. A detailed description of the results is reported in SI (Figures S21–S27 and Tables S2, S3). The initial part of the curve recorded for the titration of NaDC into water describes the endothermic demicellization process of the NaDC micelles (Figure 3). Two critical micelle concentrations denoted CMC_pre_ (5.3 mM) and CMC (8.4 mM) were obtained (Figure 3, inset). They are related to the formation of pre‐micelles and micelles of NaDC, respectively.^[19]^ The titration of NaDC solution into a solution of PNIPAM_71_ did not impact the CMC values, instead an effective CMC in the presence of the polymer (CMC*) of 6.5 mM was observed, which demonstrates that the interaction of the homopolymer with NaDC was very poor. This was also indirectly evidenced by the cryo‐TEM experiments where no supramolecular structure was found in the mixture of PNIPAM_71_ and NaDC (Figure S22).

The ITC curves of NaDC solution titrated into a PNIPAM_65_‐*b*‐PAMPTMA(+)_20_ solution presented three endothermic transition peaks (Figures 3 and S21b). One peak at low *CR* values (*Region I*, 0–0.4), which corresponds to the formation of single helices occurring via the self‐assembly of NaDC micelles onto the PAMPTMA(+) block of the copolymers. One peak at intermediate *CR* values (*Region II*, 0.4–0.9), where further formation of helices in parallel with condensation into bundles and toroids takes place. At the end of this region, corresponding to the saturation concentration *C*_s_ (=7.9 mM), the copolymer is saturated by the BS. The third peak at *CR*≥0.9 (*Region III*) describes the demicellization and the dilution of NaDC titrating micelles. The shape of the curve in this region is similar to those obtained for the NaDC titration into water or PNIPAM_71_ solution, which highlights the poor interaction between NaDC and the PNIPAM block of the copolymer (Figure 3), and a CMC* of 12 mM was found.

We also performed a titration of 30 mM NaDC solution into a low‐concentration copolymer solution (0.1 wt %). The ITC curve showed the same *CR* regions (Figure S24) in a similar *CR* range (0–2.9) as the curve obtained from the experiment using higher bile salt and copolymer concentrations (Figure 3). This demonstrates that the copolymer–BS mixing ratio plays a critical role in the formation of supramolecular helices and their condensation.

The effect of salt (NaCl) on the complex formation was also investigated (see SI). The ITC data in Figure S25 indicate that, as a result of the screened electrostatic interaction, the NaDC micellization occurred over a much narrower concentration range. Comparing the titration curves of NaDC into the block copolymer solutions without or with 50 mM NaCl, they follow the same trends (Figure S25d). From this, we may conclude that the presence of salt caused a weakening of the co‐assembly of the BS with the block copolymer, however it did not completely vanish at this salt concentration. This was also reflected by the fact that thinner bundles with smaller average width were formed in the copolymer–BS mixture with 50 mM NaCl at *CR*=1 (Figure S26).

A 200 mM NaDC solution was titrated into a 0.5 wt % aqueous solution of a diblock copolymer with the same PAMPTMA(+) block length but with a shorter PNIPAM block (PNIPAM_48_‐*b*‐PAMPTMA(+)_20_). The ITC curve is displayed in Figure S27 together with that of the PNIPAM_65_‐*b*‐PAMPTMA(+)_20_–NaDC system for comparison. Similar to the PNIPAM_65_‐*b*‐PAMPTMA(+)_20_–NaDC system, the curve exhibits three endothermic peaks. The first peak in *Region I* spanned the same *CR* range as for the longer copolymer system, which further supported that this region was mainly associated to the single helix formation. The endothermic peak in *Region II* has a higher amplitude and shifts towards higher *CRs* for the shorter copolymer system demonstrating that PNIPAM plays a crucial role in the formation and stabilization the bundles.

**Figure 4:**
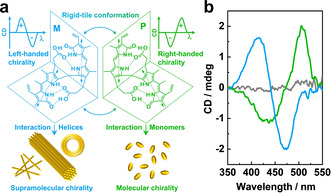
a) Schematic illustration of the interaction of two enantiomeric (*M*‐ and *P*‐)forms of BR with chiral species, generating a left‐handed and right‐handed CD signal, respectively. b) CD spectra (circular dichroism in millidegrees versus wavelength) of BR in 1 mM NaDC solution (green), in 0.1 wt % PNIPAM_48_‐*b*‐PAMPTMA(+)_20_/1 mM NaDC mixture at *CR*=0.5 (light blue), and in water (gray). The BR concentration was 100 μM.

Circular dichroism (CD) spectroscopy was utilized to demonstrate the chiral nature of the mixed complexes and at the same time examine their drug loading ability. Bilirubin‐IXα (BR), which was used as a probe, is a yellow pigment of jaundice, extensively investigated in biomedicine focusing on the pathophysiology of abnormalities in bile pigment metabolism. The molecule has two interconverting enantiomeric “ridge tile” conformations that are stabilized by six intramolecular hydrogen bonds (Figure 4 a). These molecular conformations have been experimentally observed in crystals and computationally predicted as the most stable conformations of the molecule.^[20]^ Chiral molecules and molecular aggregates can selectively interact with one of the conformations and by that determine an enantiomeric excess as revealed by a typical CD signal.

It can be observed in Figure 4 b that a mixed solution of BR and NaDC at a concentration lower than the CMC (1 mM) provided a bisignate CD cotton effect. The signal showed a negative band at a shorter wavelength (435 nm) and a positive band at a longer wavelength (506 nm) implying the right‐handed chiral signal, which in turn suggests a selective interaction of the NaDC monomer with the P‐form of BR^[20]^ (Figure 4 a). An inverted CD spectrum (left‐handed signal) with a positive band at a shorter wavelength (415 nm) and a negative band at a longer wavelength (472 nm) was observed for BR in the mixture of the PNIPAM_48_‐*b*‐PAMPTMA(+)_20_ copolymer and NaDC of the same low concentration (1 mM) (Figure 4 b), which confirms an enantioselection of the M‐form of BR by the helices. The same selectivity was observed for the interaction of BR with the single helices of the PAMPTMA(+)_130_–NaDC mixed complexes, with bundles and toroids of the other copolymer–NaDC systems, and in micellar solution of NaDC at a much larger concentration (30 mM) (Figure S28). The CD results therefore demonstrate the specific selectivity of the supramolecular helices besides a general ability of the polymer–BS complexes to load molecules.

## Conclusion

To build architectures from the bottom up by non‐covalent interactions of molecular building blocks continues to be the fascinating focus of modern science aimed at fabricating reversible functional materials. Within this scenario, the herein reported reproduction of DNA condensation via hierarchical organization of small molecules unveils the unexplored ability of supramolecular self‐assembled superstructures to reproduce specific functional organization of macromolecules, thereby inspiring visionary ideas of constructing supramolecular versions of biological macromolecules. BSs like NaDC are natural amphiphiles with a specific self‐assembly[^12^, ^19a^, ^21^] that play crucial roles in biology ranging from metabolic regulation to solubilization of dietary lipids.^[22]^ The biological origin of BSs and their pharmaceutical application make the reported condensation‐similar assembly relevant to biomedical and environmentally friendly technologies. The ability to load drugs, demonstrated with bilirubin‐IXα, highlights the potential application of copolymer–BS bundles and toroids in drug encapsulation and delivery. Especially the toroidal structure is promising for providing better protection of the drug molecule with respect to stability and activity. The revealed complex chirality at molecular and supramolecular levels could be an advantage of the proposed nanocarriers to be used for a targeted chirality‐driven delivery, which is recently emerging as a true innovation in the design of efficient drug carriers.^[23]^

## Conflict of interest

The authors declare no conflict of interest.

## Supporting information

As a service to our authors and readers, this journal provides supporting information supplied by the authors. Such materials are peer reviewed and may be re‐organized for online delivery, but are not copy‐edited or typeset. Technical support issues arising from supporting information (other than missing files) should be addressed to the authors.

Supporting Information

Supporting Information

Supporting Information

Supporting Information

Supporting Information

Supporting Information

Supporting Information
