# Supplementary material for: Condensed Supramolecular Helices: The Twisted Sisters of DNA
Source: Angew Chem Int Ed Engl. 2021 Dec 3;61(4):e202113279. doi: 10.1002/anie.202113279 (PMC9300030; doi:10.1002/anie.202113279)
Supplement: Supplementary file 1 — Supporting Information [file ANIE-61-0-s003.pdf]

Angewandte Chemie
Eine Zeitschrift der Gesellschaft Deutscher Chemiker

Supporting Information

# Condensed Supramolecular Helices: The Twisted Sisters of DNA

*Guanqun Du, Domagoj Belić, Alessandra Del Giudice, Viveka Alfredsson, Anna M. Carnerup, Kaizheng Zhu, Bo Nyström, Yilin Wang, Luciano Galantini,* and Karin Schillén**

anie_202113279_sm_miscellaneous_information.pdf
anie_202113279_sm_Movie_S1.mov
anie_202113279_sm_Movie_S2.mov
anie_202113279_sm_Movie_S3.mov
anie_202113279_sm_Movie_S4.mov
anie_202113279_sm_Movie_S5.mov
anie_202113279_sm_Movie_S6.mp4

### Author Contributions

G.D. Formal analysis: Equal; Funding acquisition: Equal; Investigation: Lead; Methodology: Equal; Validation: Equal; Visualization: Lead; Writing—original draft: Lead; Writing—review & editing: Lead
D.B. Formal analysis: Supporting; Investigation: Equal; Methodology: Equal; Validation: Equal; Writing—review & editing: Equal
A.D. Investigation: Equal; Resources: Equal; Validation: Equal; Writing—review & editing: Equal
V.A. Funding acquisition: Equal; Methodology: Equal; Resources: Equal; Supervision: Supporting; Writing—review & editing: Equal
A.C. Methodology: Supporting; Resources: Equal; Validation: Equal; Writing—review & editing: Equal
K.Z. Resources: Equal; Validation: Equal; Polymer synthesis: Lead
B.N. Resources: Equal; Validation: Equal; Writing—review & editing: Equal
Y.W. Funding acquisition: Equal; Methodology: Equal; Resources: Equal; Supervision: Supporting; Validation: Equal; Writing—review & editing: Equal
L.G. Conceptualization: Equal; Formal analysis: Equal; Funding acquisition: Equal; Methodology: Equal; Resources: Equal; Supervision: Lead; Validation: Equal; Writing—original draft: Lead; Writing—review & editing: Lead
K.S. Conceptualization: Equal; Formal analysis: Equal; Funding acquisition: Lead; Methodology: Equal; Project administration: Lead; Resources: Lead; Supervision: Lead; Validation: Equal; Writing—original draft: Lead; Writing—review & editing: Lead.

SUPPORTING INFORMATION

## Table of Contents


1. **Materials and Methods** ... 3

   1.1 **Materials and sample preparation** ... 3

   1.2 **Methods** ... 3

      1.2.1 Cryogenic transmission electron microscopy ... 3

      1.2.2 Cryogenic electron tomography ... 3

      1.2.3 Small and wide-angle X-ray scattering ... 4

      1.2.4 Circular dichroism ... 4

      1.2.5 Proton nuclear magnetic resonance spectroscopy ... 4

      1.2.6 Electrophoretic light scattering ... 4

      1.2.7 High-sensitivity isothermal titration calorimetry ... 4

2. **Results and Discussion** ... 5

   2.1 **Supramolecular polyelectrolyte-bile salt complexes** ... 5

   2.2 **Supramolecular block copolymer-bile salt complexes – bundles and toroids** ... 7

      2.2.1 Cryo-TEM experiments on $PNIPAM_m$-*b*-$PAMPTMA(+)_{20}$-NaDC mixed complexes ... 7

      2.2.2 Cryo-ET experiments on $PNIPAM_{65}$-*b*-$PAMPTMA(+)_{20}$-NaDC mixed complexes ... 10

      2.2.3 Cryo-TEM and cryo-ET on $MPEG_{45}$-*b*-$PAMPTMA(+)_{21}$-NaDC mixed complexes ... 11

   2.3 **Helical structure and hexagonal packing evidenced by SAXS/WAXS** ... 14

   2.4 **NMR experiments on hexagonally packed supramolecular helices** ... 15

   2.5 **Thermodynamics of mixed complex formation** ... 16

      2.5.1 Dilution of NaDC micellar solution ... 16

      2.5.2 $PNIPAM_{71}$ homopolymer-NaDC system ... 17

      2.5.3 $PNIPAM_{65}$-*b*-$PAMPTMA(+)_{20}$ block copolymer-NaDC system ... 17

      2.5.4 Salt effect ... 19

      2.5.5 $PNIPAM_{48}$-*b*-$PAMPTMA(+)_{20}$ block copolymer-NaDC system ... 20

   2.6 **Chirality recognition of hexagonally packed supramolecular helices** ... 20

**Movie Captions** ... 21

**Reference** ... 21

**Author Contributions** ... 22

# 1. Materials and Methods

## 1.1 Materials and sample preparation

Poly(*N*-isopropylacrylamide)$_m$-*block*-poly(3-acrylamidopropyl) trimethylammonium chloride)$_{20}$ (PNIPAM$_m$-*b*-PAMPTMA(+)$_{20}$) diblock copolymers of narrow molecular weight distributions and values of $m$ = 65 or 48, corresponding to nominal molar masses ($M$) of 11591 g mol$^{-1}$ and 9670 g mol$^{-1}$, respectively. Details about the synthesis and characterization of the polymers are given in ref.[1] The polymerization degrees and the $M$ values were determined by proton nuclear magnetic resonance ($^1$H NMR). The synthesis procedures of the homopolymers, poly(N-isopropylacrylamide) (PNIPAM$_{71}$, $M$ = 8139 g mol$^{-1}$) and poly(3-acrylamidopropyl) trimethylammonium chloride (PAMPTMA(+)$_{130}$, $M$ = 27069 g mol$^{-1}$) are reported in ref.[1-2] The diblock copolymer methoxy-poly(ethylene glycol)-*block*-poly((3-acrylamidopropyl) trimethyl ammonium chloride) here abbreviated as MPEG$_{45}$-*b*-PAMPTMA(+)$_{21}$ ($M$ = 6432.5 g mol$^{-1}$) (number-average molar mass $M_n$ = 7.87 × 10$^3$, and polydispersity index $M_w/M_n$ = 1.12) was synthesized according to our previously reported procedure.[3] Bile salt (BS) sodium deoxycholate (NaDC, purity ≥ 98%, $M$ = 414.55 g mol$^{-1}$) was purchased from Sigma-Aldrich. Bilirubin IXα (purity = 99%, $M$ = 584.67 g mol$^{-1}$) was purchased from J&K (Beijing, China). All chemicals were used without further purification. Water purified by a Milli-Q system (Millipore Corporation, Bedford, MA) equipped with a sterile 0.22 µm membrane filter was used as the solvent.

The aqueous stock solutions of PNIPAM$_m$-*b*-PAMPTMA(+)$_{20}$ and MPEG$_{45}$-*b*-PAMPTMA(+)$_{21}$ block copolymers were prepared by weighing and stirred at 5 – 6°C for 24 h, whereas the stock solutions of NaDC and PAMPTMA(+)$_{130}$ were equilibrated at room temperature. Prior to use the block copolymer stock solutions were equilibrated at room temperature for about 30 min. The mixed solutions were prepared from the stock solutions (“BS solution into polymer solution”) at a constant polymer concentration of either 0.10 wt% or 0.50 wt% (from here on 0.1 and 0.5 wt%) depending on the technique. The negative-to-positive charge ratio $CR = n_-/n_+$, where $n_-$ and $n_+$ are the number of moles of negative charge (= number of moles of BS) and positive charge (= polymerization degree of PAMPTMA(+) block × number of moles of the polymers) varied from 0 to 4 ($CR$ = 0 denotes the pure polymer). The mixed solutions of block copolymer and BS were stirred at 5 – 6°C for 24 h before further use.

## 1.2 Methods

### 1.2.1 Cryogenic transmission electron microscopy

Cryogenic transmission electron microscopy (Cryo-TEM) experiments were carried out on a JEM-2200FS transmission electron microscope (JEOL) specially optimized for cryo-TEM at the National Center for High Resolution Electron Microscopy (nCHREM) at Lund University. The microscope has a field-emission electron source, a cryo pole piece in the objective lens and an in-column energy filter (omega filter). Specimens were prepared using Leica EM GP automatic plunge freezer system from Leica Microsystems, Stockholm, Sweden with the environmental chamber set to 21°C and with relative humidity 90 %. A 4 µL droplet of the sample solution was deposited on a lacey formvar carbon coated grid (Ted Pella), which had been glow-discharged in a Quorum GloCube system (Quorum Technologies, Laughton, UK), and was blotted with filter paper to remove excess fluid. The grid was then plunged into liquid ethane (around -183°C) to ensure rapid vitrification of the sample in its native state. The specimens were thereafter stored in liquid nitrogen (-196°C). Prior to the experiments, they were transferred into microscope using a cryo-transfer tomography holder (Fischione, Model 2550). A bottom-mounted TemCam-F416 camera (TVIPS) using SerialEM[4] recorded zeroloss images under low-dose conditions mode (total electron dose per acquired image < 30 electrons/Å$^2$) at an acceleration voltage of 200 kV. The sample temperature was kept below -174°C during imaging. The acquired cryo-TEM images were processed using ImageJ/Fiji software.[5] The polymer concentration of the samples was 0.1 wt%.

### 1.2.2 Cryogenic electron tomography

Cryogenic electron tomography (Cryo-ET) measurements were also performed to further investigate the co-assembly of the supramolecular structures in three dimensions. For this purpose, 10 or 20 nm Au fiducial markers (UMC Utrecht or Sigma-Aldrich, respectively) were added to the block copolymer-BS mixtures to aid in the alignment of the electron tomography data set. PNIPAM$_{65}$-*b*-PAMPTMA(+)$_{20}$/NaDC mixed solution at $CR$ = 1 with 0.1 wt% copolymer concentration was chosen as a suitable and representative sample. The vitrification protocol for cryo-ET was the same as for cryo-TEM. Initial assessment of the cryo-ET samples confirmed that the addition of Au fiducials had no observable effect on the polymer-BS supramolecular structures: except for the presence of Au nanoparticles, the cryo-ET samples appeared identical to the pristine samples prepared for cryo-TEM. A series of 2D cryo-TEM images of the same area were collected at tilt angles from -60° to +60° at a nominal defocus between -1.5 and -3 µm using SerialEM software

package. Typically, we recorded a total of 61 images in 2° increments, keeping the electron dose per tilt view < 1.5 electrons/Å$^2$, i.e., the total electron dose per imaged area < 120 electrons/Å$^2$. For comparison, the polymer-BS supramolecular structures proved resilient against electron beam damage up to 500 electrons/Å$^2$, even when a noticeable degradation of the vitreous ice near the supporting carbon film was observed. Tilt series processing and 3D reconstruction were performed using the IMOD software package.[6] The tomographic reconstructions (typically 2048 × 2048 × 500 voxels) were done using a simultaneous iterative reconstruction technique (SIRT) algorithm after 15 iterations. Data visualization was performed using IMOD, ImageJ/Fiji and Chimera.[7]

### 1.2.3 Small and wide-angle X-ray scattering

Small and wide-angle X-ray scattering (SAXS/WAXS) experiments were performed on a Saxslab Ganesha instrument, equipped with JJ X-ray system Aps pinhole, an X-ray microsource with a wavelength $\lambda$ = 1.5406 Å, and a movable two-dimensional 300k Pilatus detector (Dectris Ltd, Switzerland). Three detector sample-to-detector distances were used for data collection and the azimuthally averaged scattering intensities, I($q$), as a function of the scattering vector $q = (4\pi/\lambda)\sin(\theta/2)$ where $\theta$ is the angle between the scattered beam and the incoming beam. Measured scattering intensities of the solutions were subtracted by the contribution of the solvent and were put to absolute scale using a built-in calibration procedure of the instrument. All experiments were performed at 25°C. The peaks in the scattering curves were interpreted as Bragg peaks of the mesophase and lattice and reflections generated by the supramolecular co-assembled structures. The dilute mixed solutions were measured in a capillary. In some cases, in order to increase the scattering intensity, the solutions were concentrated by centrifugation for 1 – 2 h at 7000 rpm where after the denser bottom phase was collected and measured in a sandwich plate using mica as the window.

### 1.2.4 Circular dichroism

The polymers-BS mixed solutions used for the circular dichroism (CD) measurements had a polymer concentration of 0.1 wt% and contained 100 μM Bilirubin IXα (BR). The samples were prepared by adding minute volume of 5 mM stock solution of BR in dimethyl sulfoxide (DMSO) to the mixed solution. The addition corresponded to a 2 % increase in volume and thus negligible in terms of stoichiometry. The CD spectra were recorded on a JASCO J-715 CD spectrometer at 25°C with the settings of 2 nm band width, 1 s response time, 100 nm min$^{-1}$ scan rate, and 0.2 nm data pitch. The measurements were performed in the wavelength range 300 – 600 nm. Quartz cuvettes with 1 mm path length were used to keep the photomultiplier voltage below 800 V in the entire wavelength range. The presented spectra are the average of 5 scans.

### 1.2.5 Proton nuclear magnetic resonance spectroscopy

$^1$H NMR spectroscopy experiments were performed on Bruker Avance 400 MHz instrument for 10.34 mM NaDC solution, 0.5 wt % PNIPAM$_{48}$-*b*-PAMPTMA(+)$_{20}$ solution, and the PNIPAM$_{48}$-*b*-PAMPTMA(+)$_{20}$-NaDC mixed solution at *CR* = 1. The NaDC concentration was the same in both the pure and the mixed solution. $D_2O$ was used as the solvent and the measurements were carried out at 25°C. The pulse program was zg30 with 64 number of scans and a recycle delay time equal to 3 s.

### 1.2.6 Electrophoretic light scattering

Electrophoretic light scattering measurements were performed 24°C and at a scattering angle of 13° using a Zetasizer Nano ZS instrument from Malvern Instruments (Malvern Panalytical), Ltd., Malvern, U.K.[8] The mixed solutions were filtered through 0.45 μm-Millex-HV syringe filters from Merck Millipore and filled in disposable folded capillary cells.

### 1.2.7 High-sensitivity isothermal titration calorimetry

The ITC measurements were performed utilizing a TAM III microcalorimeter system from TA Instruments. 200 mM BS solution was titrated into a 930 µL sample cell, which was pre-loaded with either 800 µL MilliQ water or 0.5 wt% polymer solutions, while the reference cell contained 865 µL MilliQ water. Aliquots of concentrated surfactant solution of 3 µL were consecutively injected into the sample cell using a 500 µL Hamilton syringe controlled by a Thermometric 612 Lund pump until the desired concentration range had been covered. The solution in the sample cell was stirred at 90 rpm with a gold propeller throughout the experiments. Each injection lasted 10 s and there was an interval of 600 s between successive injections for the equilibrium. All the titration experiments were carried out at 25.00°C. The observed enthalpy change ($\Delta H_{obs}$) was obtained by integrating the peak areas in the plot of heat flow against time.

## 2. Results and Discussion

### 2.1 Supramolecular polyelectrolyte-bile salt complexes

NaDC can form gels with increasing concentration, lowering the solution pH, or by addition of salt or polyamidoamino acids.[9] Dry fibers, consisting of NaDC molecules in a helical arrangement, can be drawn from such gels as reflected in a very typical X-ray pattern.[9a, 10] This led us to perform wide angle X-ray scattering (WAXS) measurements on PAMPTMA(+)$_{130}$-NaDC mixtures at *CR* = 0.5 (concentrated) and 1 (precipitate) (Figure 1b). Both samples presented high-*q* peaks at *q* = 9.0, 10.0, 10.4, and 11.6 nm$^{-1}$, corresponding to the spacing of 0.70, 0.63, 0.60, and 0.54 nm, respectively. Sets of spacings of 0.765, 0.66, and 0.58 nm or 0.87, 0.74, 0.65, 0.58, and 0.35 nm were found by Rich and Blow[10b] and D'Archivio et. al.,[9a] which were in agreement with those measured by WAXS considering the isotropic distribution of the helices in the sample and the structural changes related to the hydration. The pattern was also appreciated in dilute solution (Figure 1c, inset), where the reflection at 9.7 nm$^{-1}$ related to the main spacing at 0.65 nm. Different structures have been proposed for the NaDC helix in the dry fiber. According to Rich and Blow, the helix unit is composed of four steroid molecules and twelve water molecules that generate the helix through a rotation of 125° and translation of 0.225 nm.[10b] In the most recent model, 8-fold helix has been suggested, which is generated by a trimer as a repetitive unit through a rotation of 360°/8 = 45° and a translation of 0.65 nm.[9a]

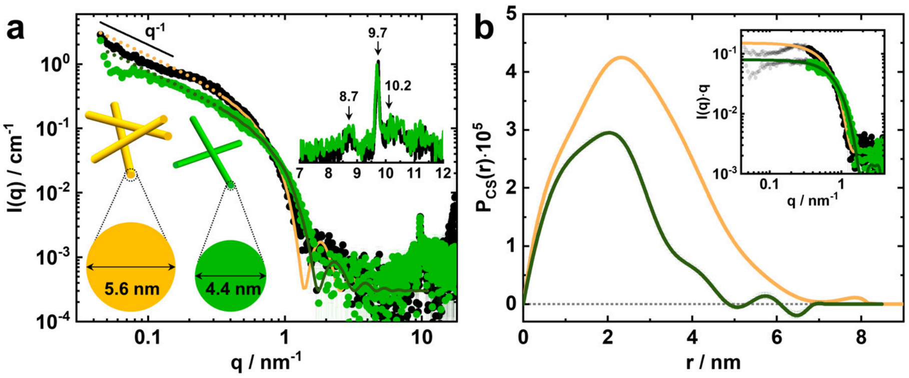


**Figure S1.** a) Comparison between the SAXS profiles of PAMPTMA(+)$_{130}$-NaDC mixture at *CR* = 0.5 with homopolymer concentration of 0.5 wt% and NaDC of 12 mM (black symbols) and of a NaDC gel solution at concentration of 20 mM, obtained by solubilization in $NaH_2PO_4$ 20 mM with final pH 6.7 (green symbols). The theoretical intensity profiles for long rods with cross section diameter of 5.6 nm (orange line for PAMPTMA(+)$_{130}$-NaDC mixture) and 4.4 nm (dark green line for NaDC gel) are also shown, the fitting starts from $q$ > 0.35 nm$^{-1}$. The power law $q^{-1}$ indicates rod-like particle. The peaks observed in the WAXS regime for both samples are indicated in the inset. b) Pair distance distribution functions of the cross section ($P_{CS}(r)$) of rod-like objects obtained by applying the indirect Fourier transform (IFT) method to the I($q$)·$q$ data starting from $q$ > 0.35 nm$^{-1}$. The corresponding fit to the experimental data is presented in the inset. The color code is the same as in (a).

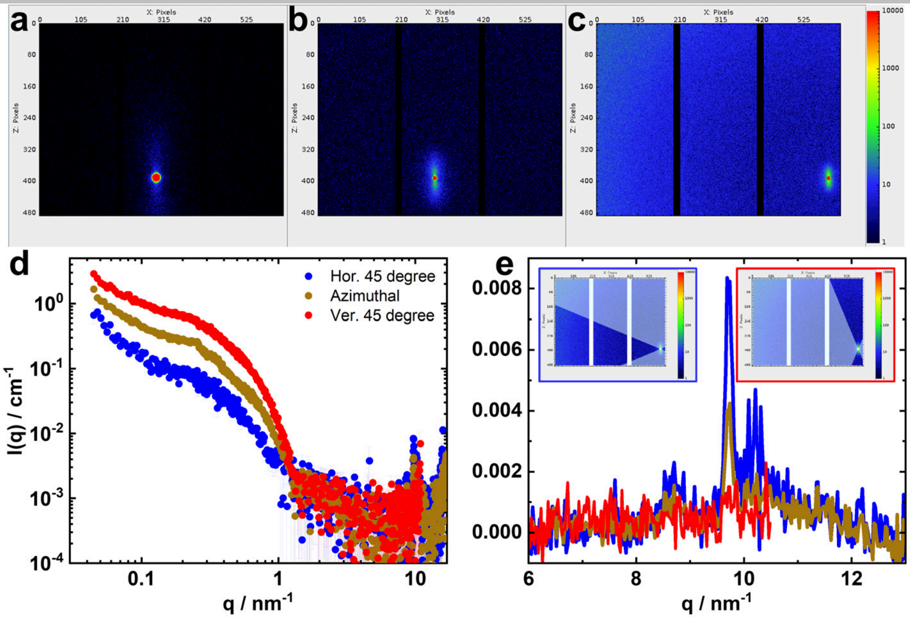


**Figure S2.** 2D detector images showing the non-isotropic intensity distribution in SAXS experiments on PAMPTMA(+)$_{130}$-NaDC mixture at *CR* = 0.5, with homopolymer concentration 0.5 wt%, placed in a horizontal capillary cell. a) Sample-detector distance 2500 mm, b) 550 mm, c) 262 mm. Comparison of azimuthally integrated data on the full available azimuthal angle (brown line) and on restricted sectors of 45° oriented parallel (blue line) or perpendicular (red line) to the capillary axis. d) Full *q* range, e) Magnification on the diffraction signals observed in the WAXS range. The sectors are schematically shown in the insets of (e).

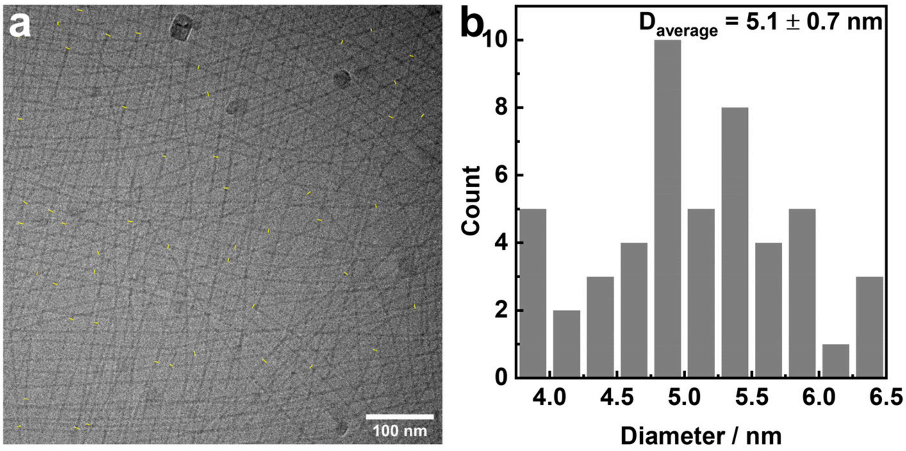


**Figure S3.** a) Cryo-TEM image of PAMPTMA(+)$_{130}$-NaDC mixture at *CR* = 0.5. The diameters of the nanowires (helices) are marked with yellow lines. b) The average diameter of the helices in (a) was estimated by the count distribution of the diameters to be 5.1 ± 0.7 nm. The homopolymer concentration was 0.5 wt%.

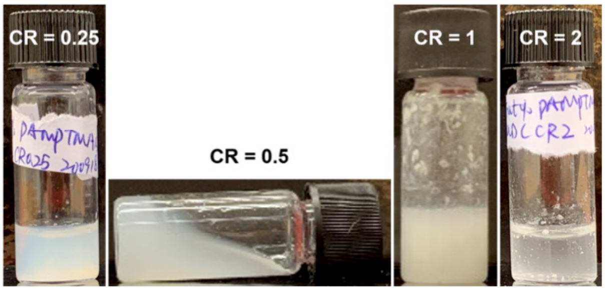


**Figure S4.** Phase behavior of the PAMPTMA(+)$_{130}$-NaDC system at *CR* = 0.25, 0.5, 1 and 2. A visual inspection of the samples shows that the *CR* = 0.25 and 0.5 samples are in the one phase region, *CR* = 1 is phase separated into a liquid dilute phase and a solid precipitate. At *CR* = 2 the system is still phase separated, however some of the precipitate start to be re-solubilized by NaDC.

## 2.2 Supramolecular block copolymer-bile salt complexes – bundles and toroids

### 2.2.1 Cryo-TEM experiments on PNIPAM$_m$-*b*-PAMPTMA(+)$_{20}$-NaDC mixed complexes

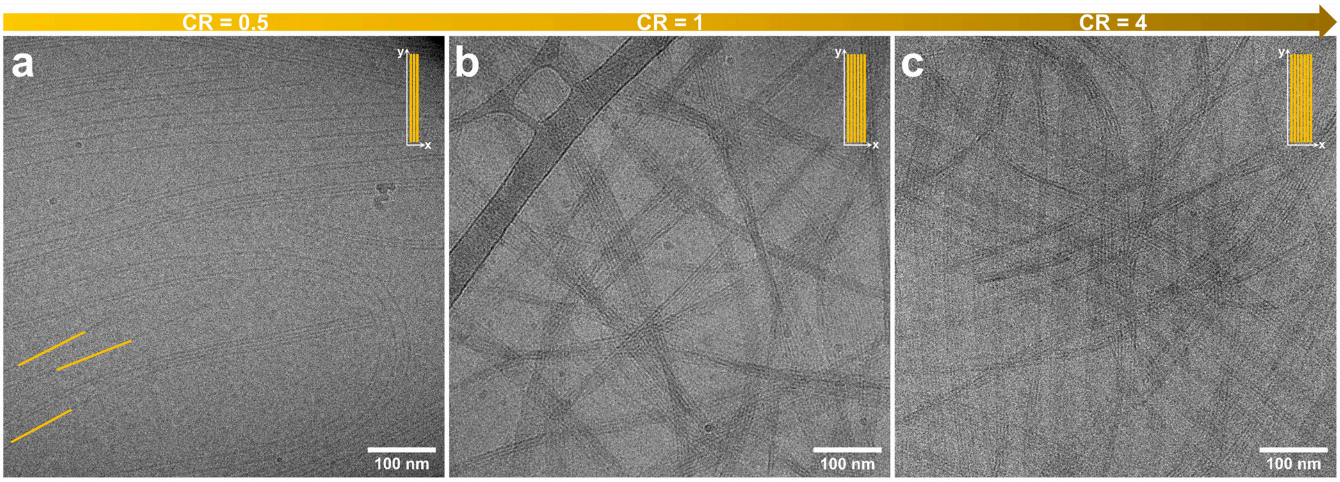


**Figure S5.** Cryo-TEM images of PNIPAM$_{48}$-*b*-PAMPTMA(+)$_{20}$-NaDC mixtures at (a) *CR* = 0.5, (b) 1, and (c) 4. Orange parallel lines in the inset of each image show the growth in width of the bundles. Single helices are highlighted in (a). The copolymer concentration was 0.1 wt%.

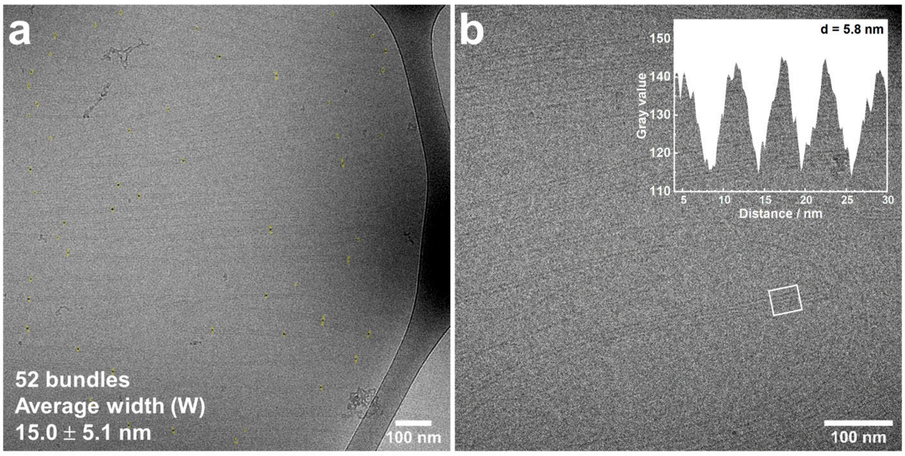


**Figure S6.** Cryo-TEM on PNIPAM$_{48}$-*b*-PAMPTMA(+)$_{20}$-NaDC mixed complexes at *CR* = 0.5. a) Analysis of the bundle width (yellow lines). b) Periodic distance (5.8 nm) in the bundle estimated from gray value analysis (inset) for the marked region. The copolymer concentration was 0.1 wt%.

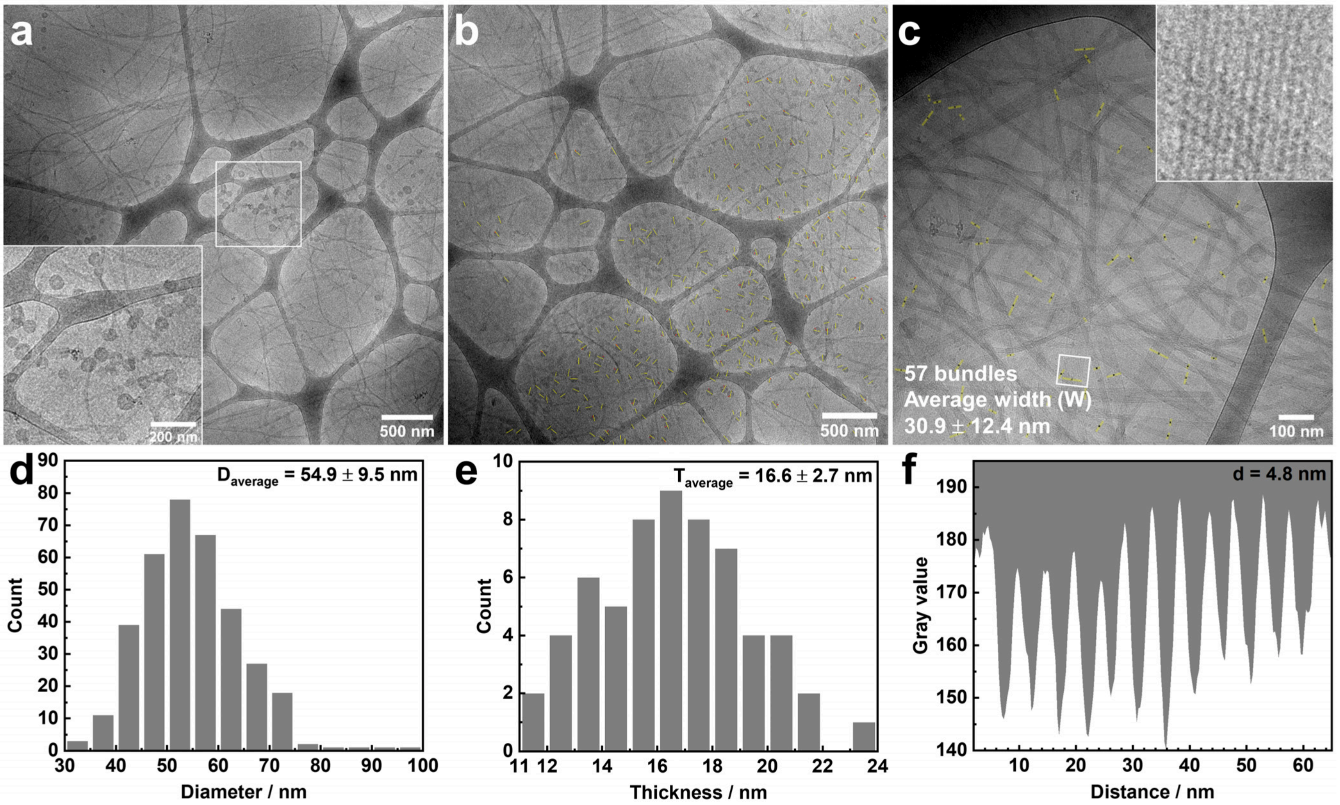


**Figure S7.** Cryo-TEM on PNIPAM$_{48}$-*b*-PAMPTMA(+)$_{20}$-NaDC mixed complexes at *CR* = 1. a) Representative overview image with the enlarged region showing bundles and toroidal structures. b) Size analysis of the toroidal structure with corresponding count distributions of the diameter (yellow lines) in (d) and wall thickness (red lines) in (e). c) Bundle width analysis (yellow lines) and the periodic distance (4.8 nm) estimation from gray value analysis in (f) for the marked and enlarged region (inset). The copolymer concentration was 0.1 wt%.

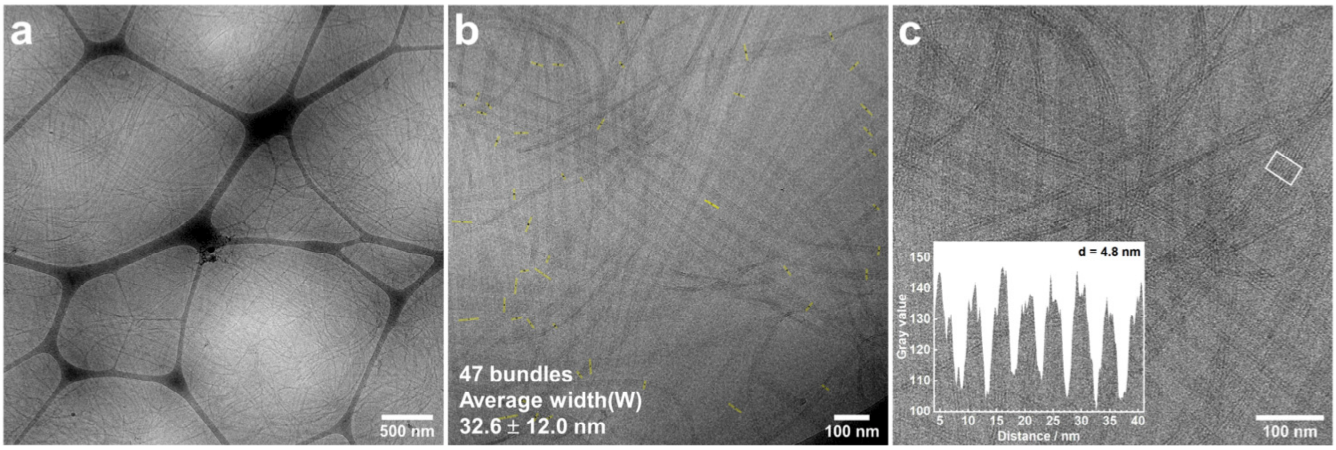


**Figure S8.** Cryo-TEM on PNIPAM$_{48}$-*b*-PAMPTMA(+)$_{20}$-NaDC mixed complexes at *CR* = 4. a) Representative overview image of the bundles. b) Bundles and analysis of bundle width (yellow lines). c) Periodic distance (4.8 nm) estimated from gray value analysis (inset) for the marked region. The copolymer concentration was 0.1 wt%.

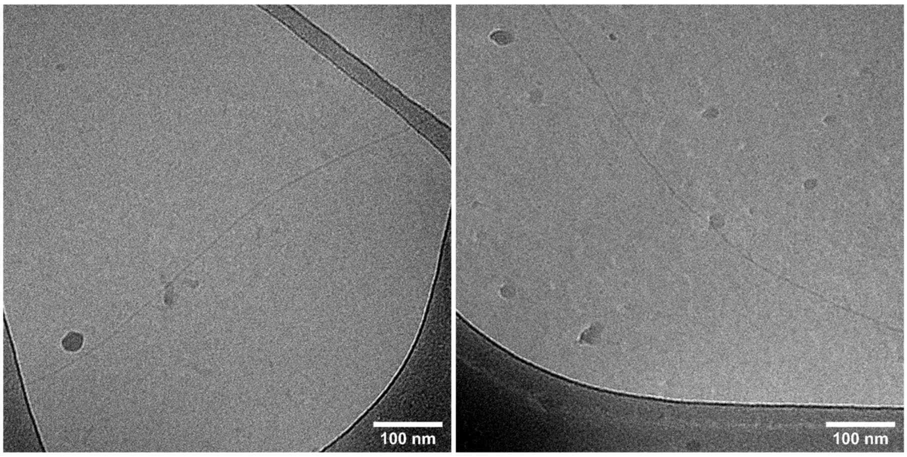


**Figure S9.** Cryo-TEM on single helices in the PNIPAM$_{65}$-*b*-PAMPTMA(+)$_{20}$-NaDC mixture at *CR* = 0.25. The copolymer concentration was 0.1 wt%.

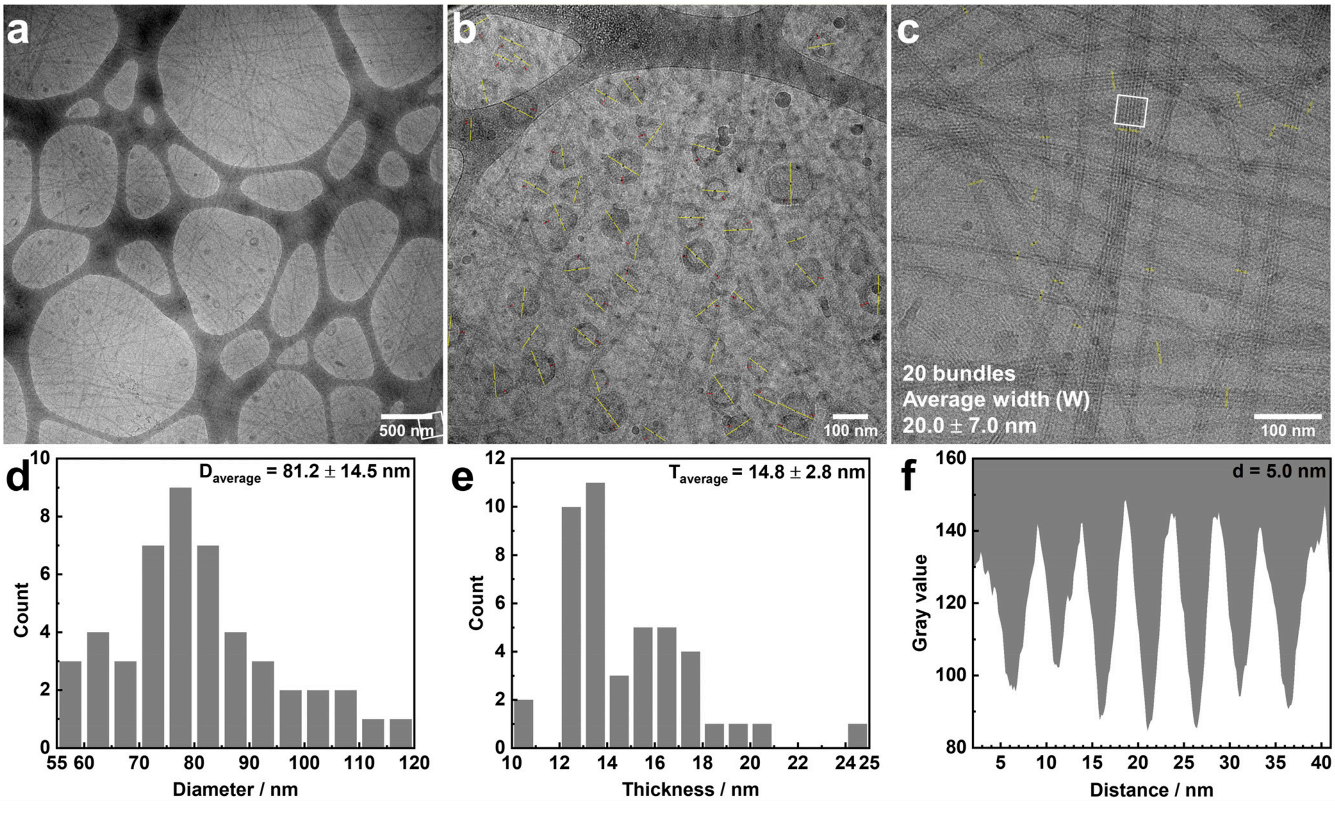


**Figure S10.** Cryo-TEM on PNIPAM$_{65}$-*b*-PAMPTMA(+)$_{20}$-NaDC mixed complexes at *CR* = 0.5. a) Representative overview image showing bundles and toroidal structures. b) Size analysis of the toroidal structure with corresponding count distributions of the diameter (yellow lines) in (d) and wall thickness (red lines) in (e). c) Bundle width analysis (yellow lines) and the periodic distance (5.0 nm) estimated from gray value analysis in (f) for the marked region. The copolymer concentration was 0.1 wt%.

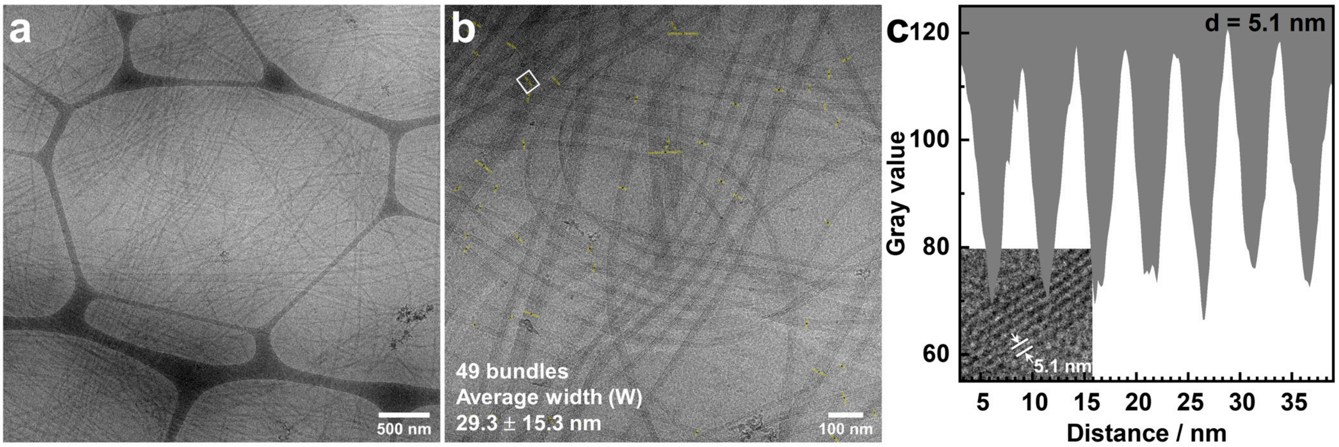


**Figure S11.** Cryo-TEM on PNIPAM$_{65}$-*b*-PAMPTMA(+)$_{20}$-NaDC mixed complexes at *CR* = 1. a) Representative overview image of the bundles. b) Analysis of bundle width (yellow lines). c) The periodic distance (5.1 nm) estimated from gray value analysis for the marked region in (b). The copolymer concentration was 0.1 wt%.

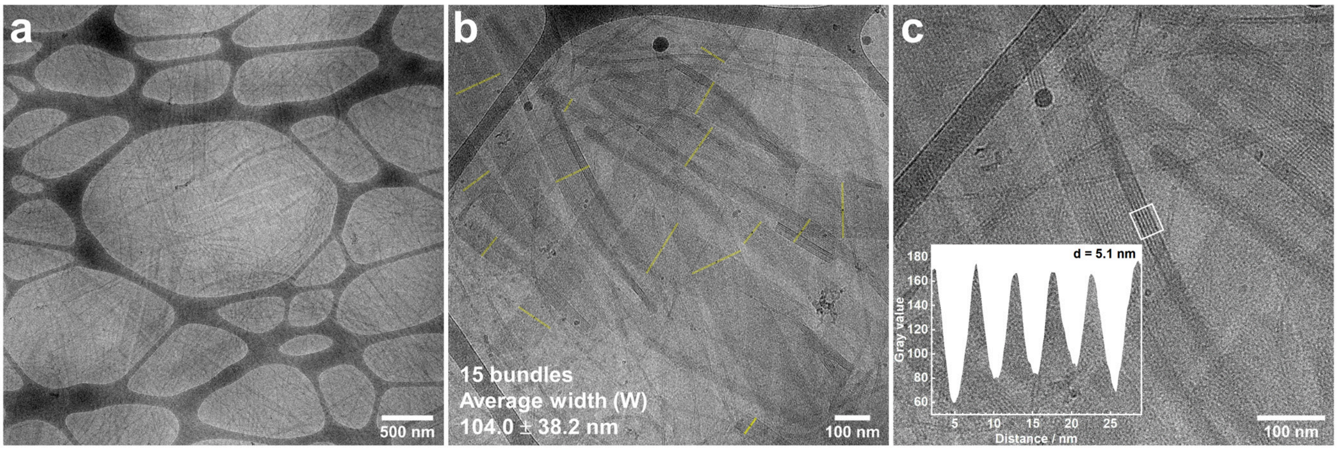


**Figure S12.** Cryo-TEM on PNIPAM$_{65}$-*b*-PAMPTMA(+)$_{20}$-NaDC mixed complexes at *CR* = 4. a) Representative overview image of the bundles. b) Bundle width analysis (yellow lines). c) Gray value analysis (inset) for the marked region for estimating the periodic distance (5.1 nm). The copolymer concentration was 0.1 wt%.

### 2.2.2 Cryo-ET experiments on PNIPAM$_{65}$-*b*-PAMPTMA(+)$_{20}$-NaDC mixed complexes

Figure S13 presents the overview image of the Cryo-ET reconstruction. The image displays 7 or 8 individual bundles of helices positioned horizontally with respect to the grid plane, while others are pictured at various tilt angles, (see also Movie S1). As can be noticed from the cross sections at different positions (marked with 1, 2 and 3), the thickness of the bundle varies along its length (Figure 1g, h and Movie S2).

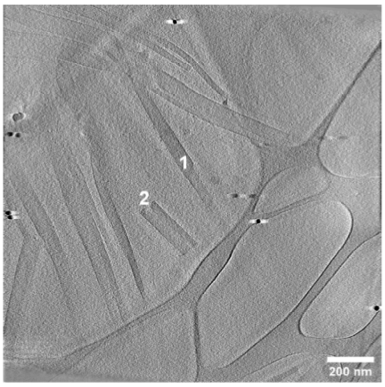


**Figure S13.** Overview of the Cryo-ET reconstruction for the bundles of the supramolecular helices in PNIPAM$_{65}$-*b*-PAMPTMA(+)$_{20}$-NaDC mixed complexes at *CR* = 1. Bundles marked with 1 and 2 were chosen as representatives for the detailed discussion. The copolymer concentration was 0.1 wt%.

Two representative bundles in Figure S13 (marked with 1 and 2) were chosen for a more detailed structural analysis. The cross sections of the bundle at different positions demonstrated a hexagonal arrangement of discrete spots (Figures 1h, S14b, and Movies S2, S3), each of them represents a single helix. A distance of 5.5 nm between two adjacent helices (inter-helix distance) and a main inter-plane distance of 4.8 nm were obtained by gray value analysis (Figure 1i). In addition, an inter-plane distance of 4.7 nm can be also estimated from another bundle with a different orientation (probably titled in 30° with respect to the image plane) (Figure S14b, c), and the corresponding inter-helix distance was calculated to be 5.4 nm (see the inserted hexagonal model in Figure S14a). The inter-helix distance or inter-plane distance could easily be resolved when one of the main planes was oriented perpendicularly (hexagonal model in Figure 1i) or parallel to the electron beam (hexagonal model in Figure S14a), respectively.

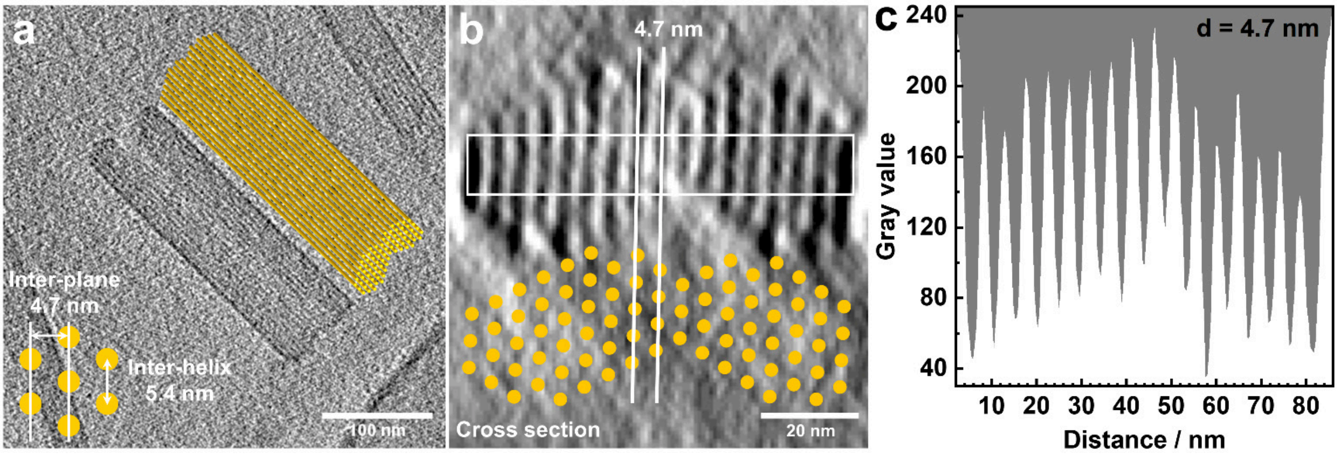


**Figure S14.** Gray values analysis of the inter-plane distance in the bundle of the $PNIPAM_{65}$-*b*-$PAMPTMA(+)_{20}$-NaDC at *CR* = 1. a) Top view and (b) cross section images of the bundle with corresponding sketched models. c) The inter-plane distance estimated for the marked region in (b). A consistent inter-plane distance of 4.7 nm is obtained.

### 2.2.3 Cryo-TEM and cryo-ET on $MPEG_{45}$-*b*-$PAMPTMA(+)_{21}$-NaDC mixed complexes

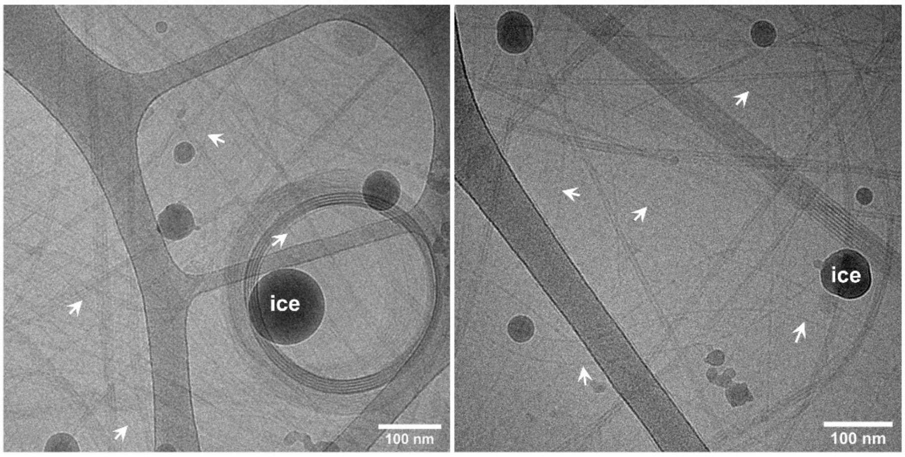


**Figure S15.** Cryo-TEM images of $MPEG_{45}$-*b*-$PAMPTMA(+)_{21}$-NaDC mixed complexes at *CR* = 0.5. The single helices are marked with arrows. The copolymer concentration was 0.1 wt%.

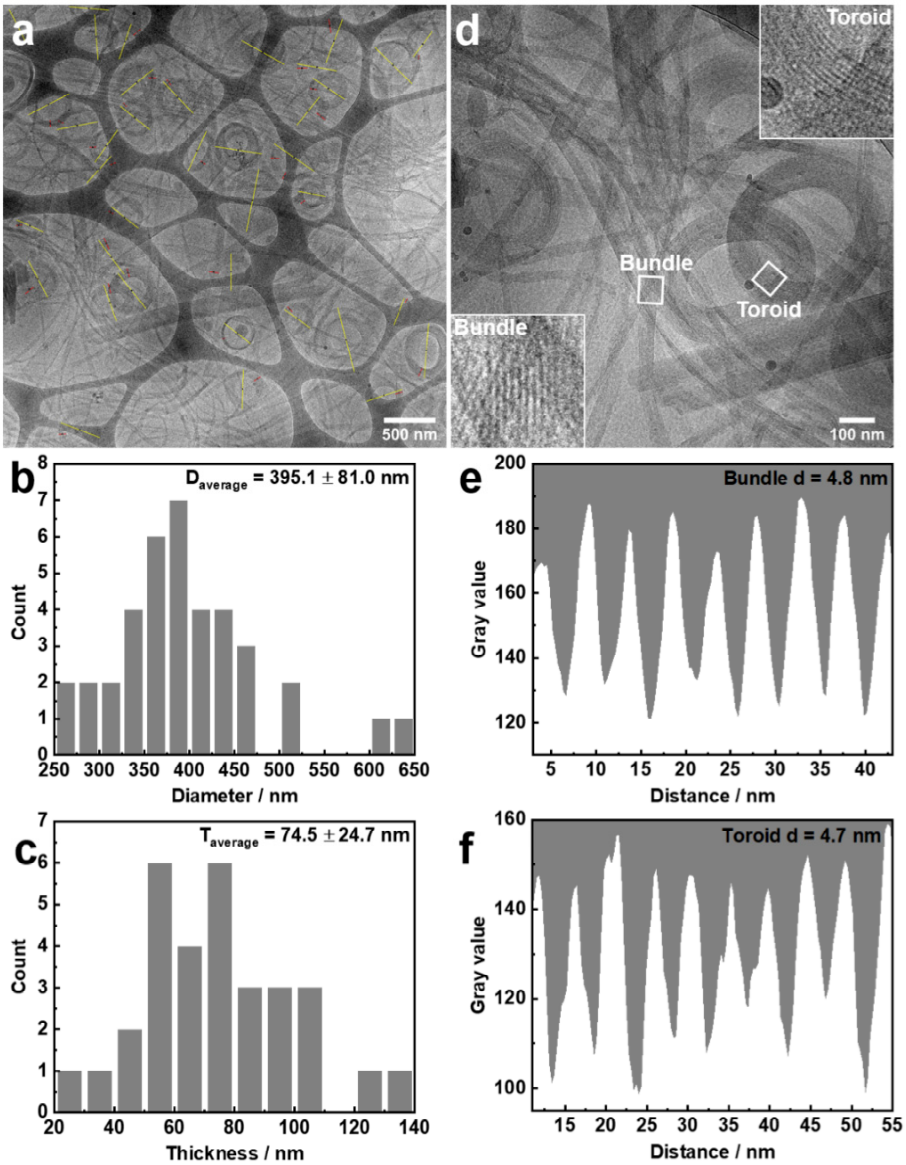


**Figure S16.** Cryo-TEM images and size analysis of MPEC$_{45}$-*b*-PAMPTMA(+)$_{21}$-NaDC mixed complexes at *CR* = 1. Size analysis of the toroidal structures in (a) with corresponding count distributions of the diameter (yellow lines) in (b) and wall thickness (red lines) in (c). e, f) Gray value analysis for the periodic distance estimation of the bundle (4.8 nm) and toroid (4.7 nm) in (d) for the marked and enlarged regions, respectively. The copolymer concentration was 0.1 wt%.

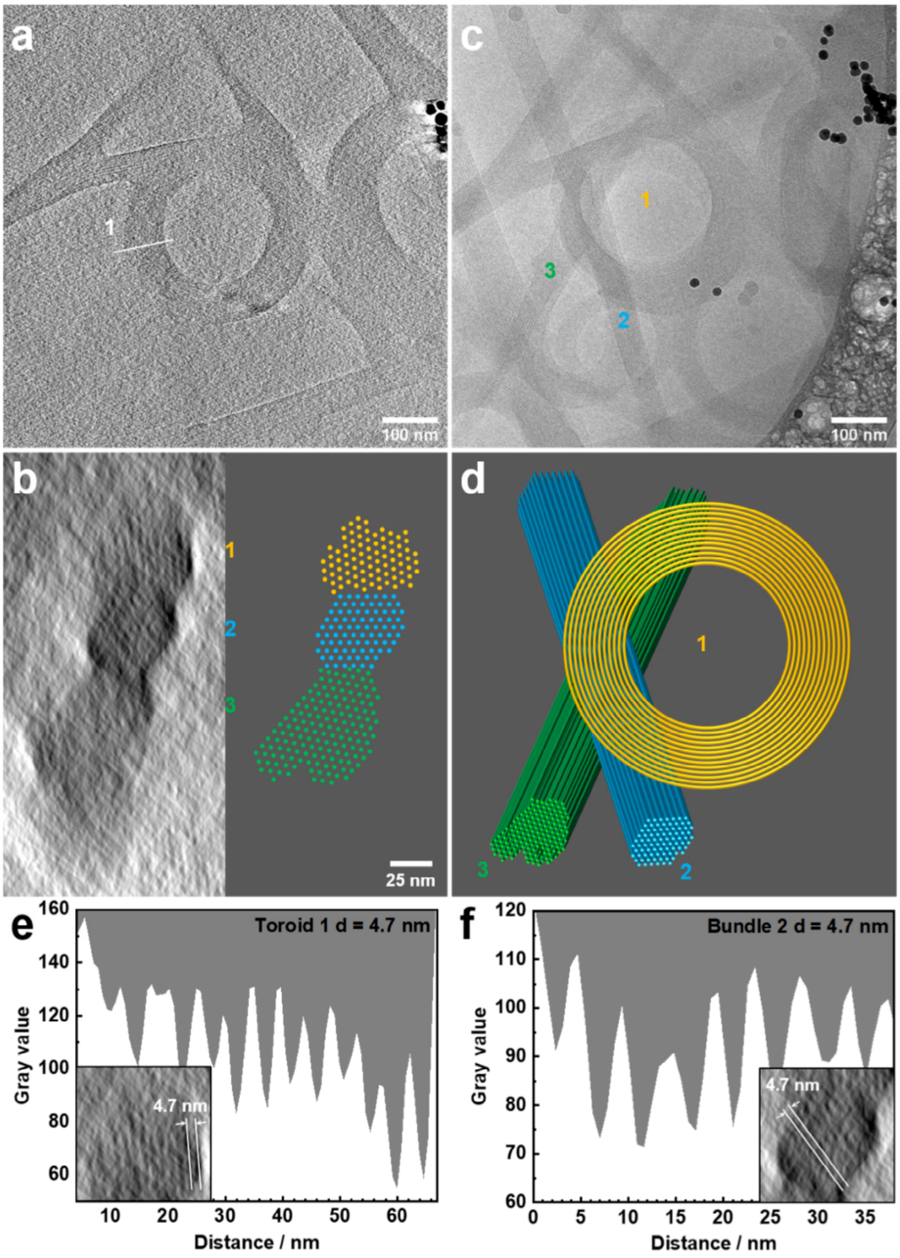


**Figure S17.** a) Cryo-ET reconstruction of MPEG$_{45}$-*b*-PAMPTMA(+)$_{21}$-NaDC mixed complexes at *CR* = 1. b) Cross sections at position 1 in (a) with corresponding sketches of three objects. c) 2D cryo-TEM image of the same area as in (a), taken after the tilt series acquisition, at this point the total electron dose sustained by this area of interest was around 200 electrons/Å$^2$, including additional adjustments and "high-resolution" image acquisition. d) 3D model sketches with suggested relative positions (orientations) of the three overlapping objects (one toroid and two bundles). e, f) Gray value analysis of the cross sections for the estimation of the inter-plane distance of the toroid 1 and bundle 2, respectively. The enlarged cross sections with the distance marked are inserted in each analysis. Color code: toroid 1 (orange), bundle 2 (blue) and bundle 3 (green). The copolymer concentration was 0.1 wt%.

Tomography measurements were performed on MPEG$_{45}$-*b*-PAMPTMA(+)$_{21}$-NaDC system at *CR* = 1, Figure S17a presents the reconstruction image of toroidal structures. The cross section at position 1 consisted of three objects piled onto each other (Figure S17b), one toroid and two bundles as indicated by the 2D cryo-TEM image of the same area (Figure S17c). Movies S4-S6 from reconstruction of these three objects revealed that toroid 1 was located at the top of the pile (as indicated by two Au particles in the Movies S4, S5), bundle 2 was in the middle and bundle 3 was positioned at the bottom of the pile. The relative orientations of the objects were also visualized in the 3D model sketches presented in Figure S17d. The complicated orientation of the overlapping objects made the tomography reconstruction difficult. Nevertheless, the toroid cross section still indicated a hexagonal arrangement of the NaDC helices (Figure S17b). It is suggested from the cross sections and corresponding sketches in Figure S17b, that one of the planes of the hexagonal array in the cross section of toroid 1 was oriented more parallel to the electron beam while it was oriented more perpendicular in bundle 2. This explains why the short vertical fringes can be observed in the cross section of the toroidal structure. It is difficult to judge the orientation of bundle 3 because of the low contrast. The same inter-plane distance (4.7 nm) was estimated by

gray value analysis of the cross sections of toroid 1 and bundle 2 (Figure S17e, f), from which an inter-helix distance of 5.4 nm was calculated. The inter-helix and inter-plane distances of the bundles formed in the MPEG$_{45}$-*b*-PAMPTMA(+)$_{21}$-NaDC system were in excellent agreement with those obtained for the PNIPAM$_m$-*b*-PAMPTMA(+)$_{20}$-NaDC systems. The effect of orientation and/or rotation on the projection of 2D images of DNA toroidal structures similar to those found in this work have been well interpreted in the literature.[11]

**Table S1.** Summary of the structural information and size analysis of the polymer-BS mixed complexes studied in this work. "Single", "Bundle", and "Toroid" represent the single helices, bundles of helices, and toroidal structure of helices, respectively. "*W*", "*D*", and "*T*" denoted for the width of the bundles, outer diameter and thickness of the toroids, respectively.

| Mixed solutions | PAMPTMA(+)$_{130}$-NaDC | PNIPAM$_{48}$-*b*-PAMPTMA(+)$_{20}$-NaDC (nm) | PNIPAM$_{65}$-*b*-PAMPTMA(+)$_{20}$-NaDC (nm) | MPEG$_{48}$-*b*-PAMPTMA(+)$_{21}$-NaDC (nm) |
|---|---|---|---|---|
| *CR* = 0.25 | - | – | Single<br><br>Bundle | – |
| *CR* = 0.5 | Single | Single<br><br>Bundle<br>*W* = 15.0 ± 5.1 | Toroid<br>*D* = 81.2 ± 14.5<br>*T* = 14.8 ± 2.8<br><br>Bundle<br>*W* = 20.0 ± 7.0 | Single<br><br>Toroid<br><br>Bundle |
| *CR* = 1 | Phase separated | Toroid<br>*D* = 54.9 ± 9.5<br>*T* = 16.6 ± 2.7<br><br>Bundle<br>*W* = 30.9 ± 12.4 | Bundle<br>*W* = 29.3 ± 15.3 | Toroid<br>*D* = 395.1 ± 81.0<br>*T* = 74.5 ± 24.7<br><br>Bundle |
| *CR* = 4 | – | Bundle<br>*W* = 32.6 ± 12.0 | Bundle<br>*W* = 104.0 ± 38.2 | – |

## 2.3 Helical structure and hexagonal packing evidenced by SAXS/WAXS

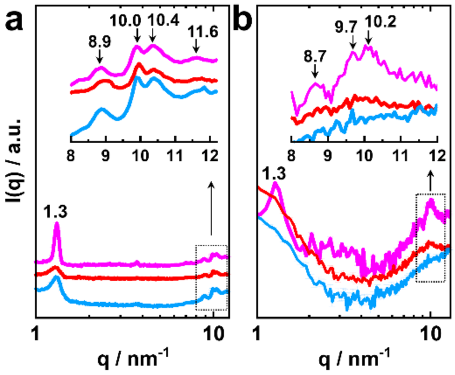


**Figure S18.** SAXS/WAXS curves of (a) mixtures (after concentrated by centrifugation) and (b) dilute mixed solutions. PNIPAM$_{48}$-*b*-PAMPTMA(+)$_{20}$-NaDC (*CR* = 4, light blue), PNIPAM$_{65}$-*b*-PAMPTMA(+)$_{20}$-NaDC (*CR* = 4, red) and MPEG$_{45}$-*b*-PAMPTMA(+)$_{21}$-NaDC (*CR* = 1, magenta). The copolymer concentration was 0.5 wt%.

The hexagonally packed helices found by cryo-ET in the copolymer-BS system were also evidenced by SAXS/WAXS. Figure S18a presents the results for the PNIPAM$_m$-*b*-PAMPTMA(+)$_{20}$-NaDC mixtures at *CR* = 4 and MPEG$_{45}$-*b*-PAMPTMA(+)$_{21}$-NaDC mixture at *CR* = 1. Due to the weak intensity of the samples, they were first concentrated by centrifugation whereafter the bottom denser phase was collected for the experiment. The SAXS curves in Figure S18a display a first order Bragg peak at $q$ = 1.3 nm$^{-1}$ from which a mean inter-plane distance (= $2\pi/q$) of 4.8 nm could be determined. An inter-helix distance of 5.5 nm was calculated assuming a hexagonal packing in good agreement with the cryo-TEM and cryo-ET results. It is important to note that the Bragg peak at $q$ = 1.3 nm$^{-1}$ was also found in the dilute mixed solutions without centrifugation, but with much lower intensity (Figure S18b). It is also interesting to mention that a hexagonal packing of the helices was also inferred from a SAXS pattern of the precipitate of the PAMPTMA$_{130}$-NaDC mixture at *CR* = 1 (Figure S19).

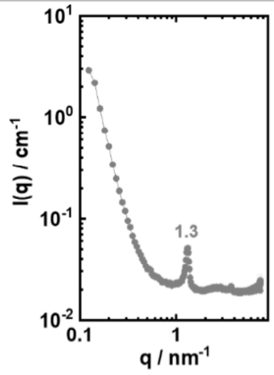


**Figure S19.** SAXS curve (I($q$) versus magnitude of the scattering vector $q$) of the homopolymer PAMPTMA(+)$_{130}$-NaDC mixture at *CR* = 1. The sample was phase separated and the curve was collected from the precipitate. The first Bragg peak at $q$ = 1.3 nm$^{-1}$ is marked. The homopolymer concentration in the initial solution was 0.5 wt%.

## 2.4 NMR experiments on hexagonally packed supramolecular helices

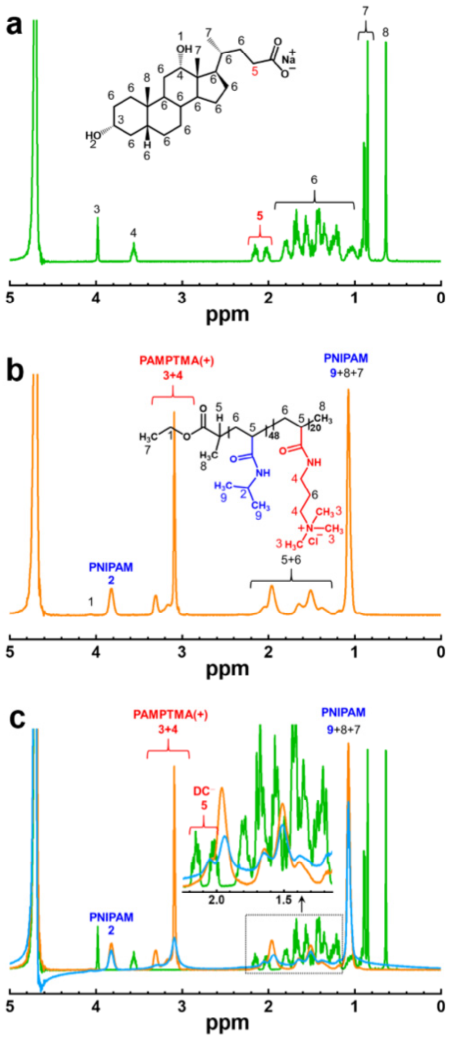


**Figure S20.** $^{1}$H NMR spectra of (a) 10.3 mM NaDC, (b) 0.5 wt% PNIPAM$_{48}$-*b*-PAMPTMA(+)$_{20}$ and (c) the mixture of PNIPAM$_{48}$-*b*-PAMPTMA(+)$_{20}$ and NaDC at *CR* = 1 (light blue), the NaDC solution (10.3 mM, green) and copolymer solution (0.5 wt%, orange) with the same concentration as in the mixture are also displayed in (c) for comparison. The marked region in (c) is enlarged in the inset. Insets of (a) and (b) show the chemical structures and assigned protons for NaDC and PNIPAM$_{48}$-*b*-PAMPTMA(+)$_{20}$, respectively. The protons of the assigned $^{1}$H resonance peaks are illustrated in each spectrum. $D_2O$ was used as the solvent.

## 2.5 Thermodynamics of mixed complex formation

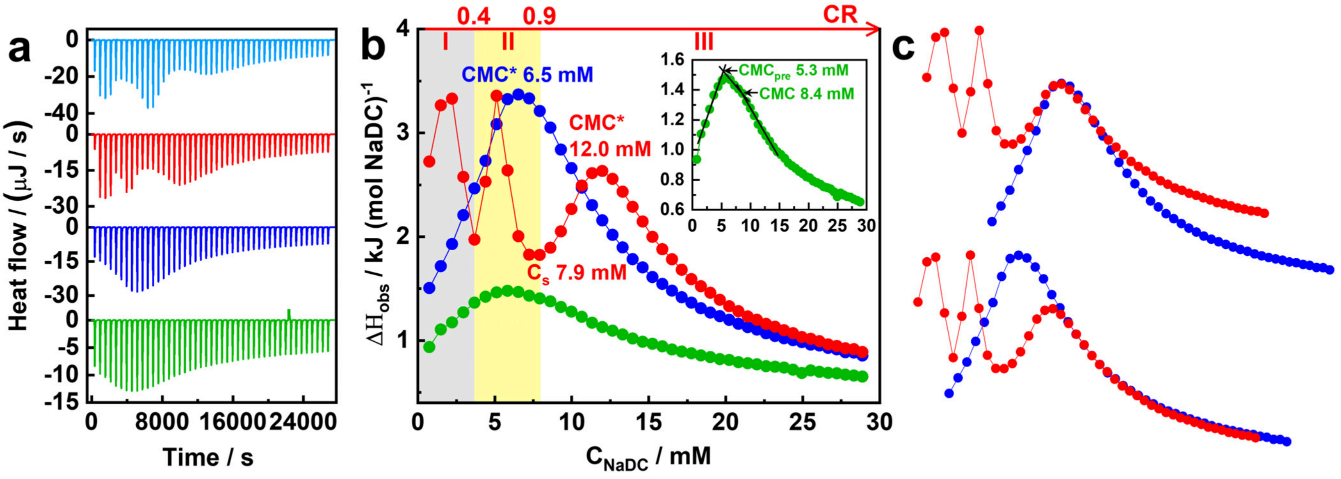


**Figure S21.** a) The raw heat flow as a function of titration time for the titration of 200 mM NaDC into water (green), $PNIPAM_{71}$ solution (blue), $PNIPAM_{65}$-*b*-$PAMPTMA(+)_{20}$ solution (red) and $PNIPAM_{48}$-*b*-$PAMPTMA(+)_{20}$ solution (light blue). b) ITC curves (observed enthalpy change $\Delta H_{obs}$ versus NaDC concentration $C_{NaDC}$) for the titration of 200 mM NaDC solution into water (green), $PNIPAM_{71}$ solution (blue) and $PNIPAM_{65}$-*b*-$PAMPTMA(+)_{20}$ solution (red). The critical micelle concentrations, $CMC_{pre}$ and CMC for the NaDC in water were determined from the intersection points of the black lines (inset) and the effective CMC* was determined from the peak values for the NaDC in the presence of polymers (see definitions in the text). c) ITC curves of $PNIPAM_{71}$-NaDC (blue) and $PNIPAM_{65}$-*b*-$PAMPTMA(+)_{20}$-NaDC (red) systems shifted to overlap in different parts of *Region III*. The incomplete overlap could be ascribed to the influence of the block copolymer-NaDC mixed complexes on the enthalpy of the demicellization process of NaDC.[12] The polymer concentration was 0.5 wt%.

### 2.5.1 Dilution of NaDC micellar solution

200 mM NaDC solution was injected into the sample cell containing MilliQ water (Figure S21b). The NaDC dilution curve displays a broad endothermic peak that has two intersection points positioned at 5.3 mM and 8.4 mM as obtained from three extrapolated lines of the curve.[13] These concentrations, denoted $CMC_{pre}$ and CMC, respectively, are related to the formation of pre-micelles and micelles of NaDC, respectively, according to the stepwise association or pseudo-phase separation model of bile salt self-assembly.[14] The small but increasingly endothermic $\Delta H_{obs}$ is related to the increase in entropy due to the hydrophobic effect, i.e., the thermodynamically unfavorable contact between non-polar groups and water, and the release of counterions during the demicellization process.[12, 15] The $\Delta H_{obs}$ values decreased and approached a plateau at high NaDC concentrations due to the dilution of the added NaDC micelles. Due to the stepwise micellization process, in addition to the difficulty of determining CMC in the temperature range where demicellization enthalpies are very small, the reported $CMC_{pre}$ and CMC of NaDC cover a wide range in the literature. We note that the values obtained in our work are in reasonable agreement with those summarized in Table S2.

**Table S2.** $CMC_{pre}$ and CMC values of NaDC determined by different physico-chemical methods reported in previous studies and in this work.

| $CMC_{pre}$ (mM) | CMC (mM) | Temperature (K) | Method | NaCl (mM) | Reference |
|---|---|---|---|---|---|
| 2.25 ± 0.12 | 10.60 ± 0.40 | 298.15 | ITC | | [16] |
| | 4.5 ± 0.2 | 298.15 ± 0.01 | ITC | | [17] |
| | 5.5 | 298 | ITC | | [18] |
| 2.4 | 6.5 | 298.2 | Static light scattering | | [19] |
| 5.3 | 8.4 | 298 | ITC | | In this work |
| | 6.4 | 303 | ITC | | [20] |
| | 10 | - | Surface tension | | [21] |
| 2.6 | 4.3 | 298 | ITC | 50 | In this work |
| 5.3 ± 0.2 | 12.3 ± 0.4 | 303 | ITC | 50 | [14a] |
| | 4.0 | 297.9 | ITC | 100 | [14d] |
| | 3 | - | Surface tension | 150 | [21] |

### 2.5.2 PNIPAM$_{71}$ homopolymer-NaDC system

The calorimetric titration curve of 200 mM NaDC solution into 0.5 wt% PNIPAM$_{71}$ homopolymer solution is presented in Figure S21b. There is no visual critical aggregation concentration (CAC) and the shape of titration curve is similar to the NaDC dilution curve but the $\Delta H_{obs}$ values are more positive in comparison. A critical concentration (rather than CAC), denoted the effective critical micelle concentration, CMC*, was obtained from the maximum of the curve. Comparing the values CMC$_{pre}$ (5.3 mM) and CMC (8.4 mM) of NaDC (inset of Figure S21b) with CMC* (6.5 mM) it is noticed that the NaDC molecules "preferred" to form free micelles instead of forming mixed micelles with the homopolymer. Hence, the intermolecular interaction between the NaDC and the low-molecular weight PNIPAM$_{71}$ can be considered to be weak.[22] The higher amplitude of the endothermic peak in the titration curve of NaDC to PNIPAM$_{71}$ compared to that of the NaDC dilution curve is likely due to small changes in the solvent environment due to the presence of PNIPAM$_{71}$ in the sample cell.[23] In addition, cryo-TEM experiment on the mixture of PNIPAM$_{71}$ and NaDC at *MR* = 0.3 (corresponds to *CR* = 1 in the PNIPAM$_{65}$-*b*-PAMPTMA(+)$_{20}$-NaDC system) did not find any supramolecular structures, which indirectly suggests there is no considerable interaction between PNIPAM$_{71}$ homopolymer and NaDC (Figure S22).

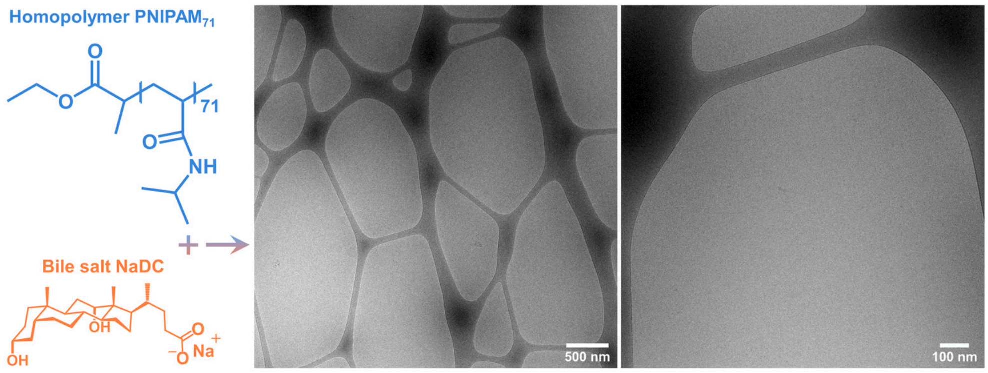


**Figure S22.** Cryo-TEM images of a mixed solution of PNIPAM$_{71}$ homopolymer and NaDC at a molar ratio (*MR*) of NaDC to NIPAM units (*MR* = n(NaDC)/[71 × n(PNIPAM$_{71}$)]). *MR* = 0.3 corresponds to *CR* = 1 in block copolymer (*m* = 65)-BS system, where *MR* = n(NaDC)/[65 × n(PNIPAM$_{65}$-*b*-PAMPTMA(+)$_{20}$)]. The n(NaDC) is the number of moles of NaDC, while n(PNIPAM$_{71}$) and n(PNIPAM$_{65}$-*b*-PAMPTMA(+)$_{20}$) are the number of moles of the homopolymer and block copolymer, respectively. The polymer concentration was 0.1 wt%.

### 2.5.3 PNIPAM$_{65}$-*b*-PAMPTMA(+)$_{20}$ block copolymer-NaDC system

200 mM NaDC was titrated into 0.5 wt% PNIPAM$_{65}$-*b*-PAMPTMA(+)$_{20}$ solution using the same settings as before. The titration curve (Figure S21b) presented three endothermic transition peaks in obvious contrast to that of NaDC into the PNIPAM$_{71}$ solution. Based on these peaks, the ITC curve was divided into three concentration (*CR*) regions marked *I*, *II* and *III*.

*Region I* (0 – 3.7 mM, *CR* 0 – 0.4) was characterized by a relatively sharp endothermic peak. The main process in this region was the cooperative association of NaDC onto PAMPTMA(+) blocks, the rearrangement of NaDC micelles and formation of single helices of DC$^-$ anions. The fact that the initial endothermic $\Delta H_{obs}$ measured for the titration of NaDC into the block copolymer solution was larger than that of the titration of NaDC into water indicated that the cooperative association starts at the initial BS addition. The entropically favorable binding process in this region was interpreted as being related to the formation of positively charged block copolymer-BS mixed complexes ($\mu$ > 0 when *CR* < 1, Table S3), in which the bound BS micelles started to rearrange to form helices at the PAMPTMA(+) blocks. The endothermic nature of the peak demonstrated that the process was entropy-driven and dominated by effects like counterion release.[24] The helices were formed at very low NaDC concentrations (or *CRs*) compared to in pure water (39.1 wt%[25]), suggesting that they were thermodynamically very stable due to the tight quasi-crystalline packing of the DC$^-$ ions in the helix. The formation of this molecular packing involved changes in the water structure, which could justify the endothermic process as well. The curve was the sum of different processes, and the continuing BS association to the single helices led to the overall declining endothermic $\Delta H_{obs}$ values after the peak maximum until 3.7 mM of NaDC, which corresponded to about *CR* = 0.4. This indicated that an optimal charge stoichiometry of the mixture for inducing the helix formation seemed to exist. It corresponded to roughly twice of the positive charge needed for neutralization, in agreement with the positive electrophoretic mobility ($\mu$ = 0.69 m$^2$/Vs × 10$^{-8}$) measured for the *CR* = 0.5 mixture (Table S3).

Figure S9 presents a cryo-TEM image of single NaDC helices, which were electrostatically stabilized by many block copolymer chains in a PNIPAM$_{65}$-*b*-PAMPTMA(+)$_{20}$-NaDC mixture at *CR* = 0.25 (corresponding to 0.43 mM NaDC). Further addition of NaDC led to larger amounts of helices formed. However, being stabilized by a lower fraction of block copolymer, they associated into bundles/toroids depending on the *CR*. This assembly or condensation process dominated *Region II* (3.7 – 7.9 mM, *CR* 0.4 – 0.9) which was characterized by the second endothermic peak. $\Delta H_{obs}$ reached a maximum value around 5 mM after which it decreased to a minimum value at 7.9 mM, denoted C$_s$ (saturation concentration). The condensation process was entropically driven due to the release of

counterions. Some dehydration of the PNIPAM blocks could also be involved in the process as the $^1$H NMR results revealed a slight restriction in the internal motion of PNIPAM part of the block copolymer chains (Figure S20).

The broad endothermic peak of *Region III* (7.9 – 28.9 mM, *CR* 0.9 – 3.8) was similar to that found in the PNIPAM$_{71}$-NaDC titration curve (Figure S21b). At this stage, the PAMPTMA(+) blocks in the complexes were saturated at $C_s$ = 7.9 mM (*CR* = 0.9) ($\mu$ = -0.09 m$^2$/Vs × 10$^{-8}$ at *CR* = 1, Table S3). At *CR* values well above 1, the exceeding NaDC micelles added could not bind to the saturated block copolymer chains. Because the interaction between NaDC and PNIPAM$_{71}$ was weak, the rising of the peak was interpreted as being related to the breakup of the titrated NaDC micelles in the environment of already formed block copolymer-BS mixed complexes. The demicellization continued until the free monomer concentration reached the CMC* (corresponding to the peak maximum), here 12.0 mM (Figure S21b) where micelles started to form. At higher NaDC concentrations, the titrated NaDC micelles were only diluted and did not break up. The latter process was accompanied by gradually decreasing $\Delta H_{obs}$ values until the titration curve merged with the PNIPAM$_{71}$-NaDC titration curve around 25 mM NaDC. As noticed in Figure S21c, the ITC curve shifted partly overlap with the PNIPAM$_{71}$-NaDC curve, which strongly supports the above point concerning a weak PNIPAM-NaDC interaction. The titration experiment was repeated, and a good reproducibility was found, see Figure S23.

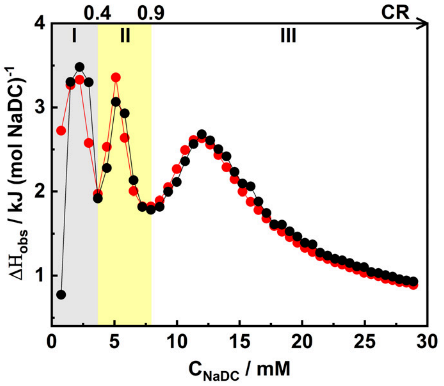


**Figure S23.** ITC curve for the titration of 200 mM NaDC solution into 0.5 wt% PNIPAM$_{65}$-*b*-PAMPTMA(+)$_{20}$ solution at 25°C (repeated experiment, black curve).

**Table S3.** Electrophoretic mobility ($\mu$) of the PNIPAM$_{65}$-*b*-PAMPTMA(+)$_{20}$-NaDC mixed solutions at different *CRs*. The copolymer concentration was 0.1 wt%.

| *CR* | $\mu$ (m²/Vs × 10$^{-8}$) |
|---|---|
| 0 | 2.45 |
| 0.5 | 0.69 |
| 1 | -0.09 |
| 4 | -0.36 |

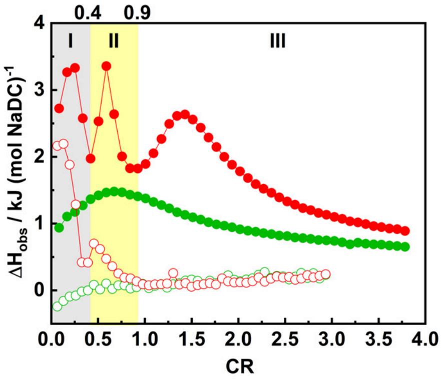


**Figure S24.** ITC curves for the titration of 30 mM NaDC micellar solution into water (green open symbols) and 0.1 wt% PNIPAM$_{65}$-*b*-PAMPTMA(+)$_{20}$ solution (red open symbols). The titration of 200 mM NaDC into water (green filled symbols) and 0.5 wt% copolymer (red filled symbols) are also presented for comparison. The

final concentration of NaDC in the ITC curves of titrating 30 mM NaDC into water or 0.1 wt% copolymer was 4.33 mM, which is below the $CMC_{pre}$ of NaDC determined in this work.

### 2.5.4 Salt effect

The ITC curves of NaDC titrated into 50 mM NaCl solution and $PNIPAM_{65}$-*b*-$PAMPTMA(+)_{20}$ solution with 50 mM NaCl and the corresponding heat flow data are displayed in Figure S25. The data in Figure S25b shows that, as a result of the screened electrostatic interaction, the NaDC micellization occurred over a much narrower concentration range in the 50 mM NaCl solution than in water. At the same time the two CMC values were lowered.[14d] The $CMC_{pre}$ and CMC of NaDC in the 50 mM NaCl solution were estimated to be 2.6 and 4.3 mM, respectively, which were about two-fold lower than in the absence of NaCl (Figure S25b, Table S2). Comparing the two titration curves of NaDC into the block copolymer solutions, it can be observed that they followed the same trends (Figure S25d). While the onset and end concentrations of *Region I* were not affected, the onset concentration of the second endothermic peak related to the helix condensation was shifted to a bit higher NaDC concentration (higher *CR*) by the addition of salt. Furthermore, the peaks had appreciably smaller amplitude in the presence of 50 mM NaCl, in particular in *Region II*. After saturation, in *Region III*, the broad endothermic peak related to the NaDC micellization, shifted towards lower concentrations (CMC* = 10.7 mM in 50 mM NaCl solution compared to 12.0 mM).

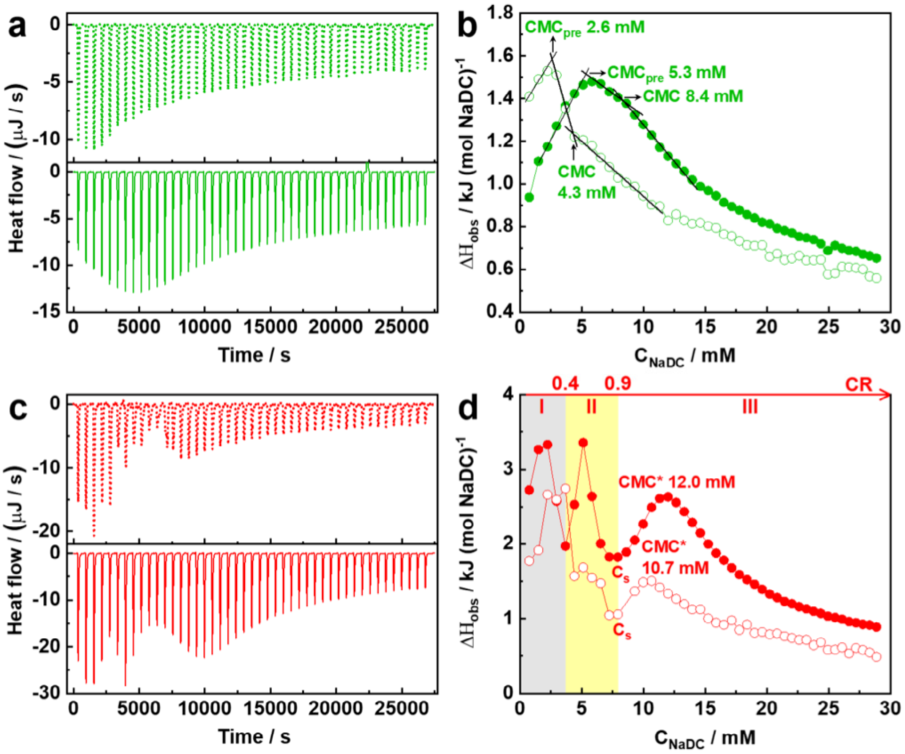


**Figure S25.** The raw heat flow as a function of titration time showing the titration of 200 mM NaDC into (a) water without (solid line) and with 50 mM NaCl (dot line) and (c) block copolymer $PNIPAM_{65}$-*b*-$PAMPTMA(+)_{20}$ without (solid line) and with 50 mM NaCl (dot line). ITC curves for the titrations of 200 mM NaDC into (b) water without (filled symbols) and with 50 mM NaCl (open symbols) and (d) block copolymer $PNIPAM_{65}$-*b*-$PAMPTMA(+)_{20}$ without (filled symbols) and with 50 mM NaCl (open symbols). The $CMC_{pre}$, CMC, and CMC* are marked in (b) and (d).

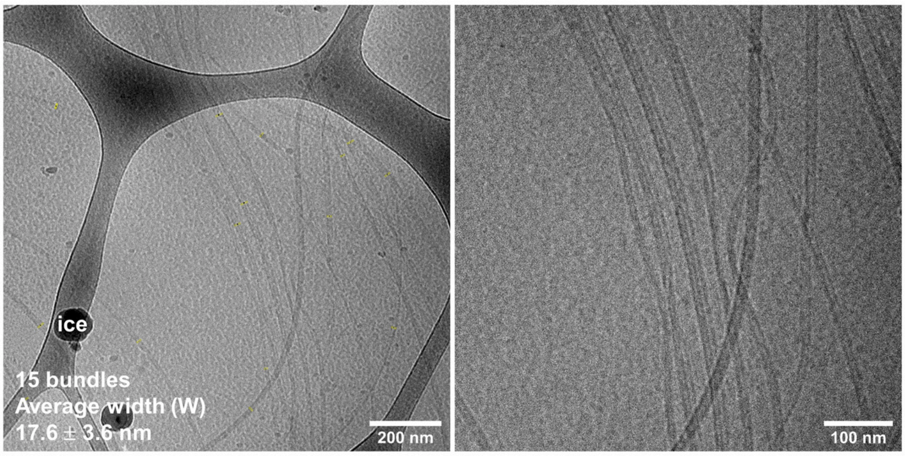


**Figure S26.** Cryo-TEM images of PNIPAM$_{65}$-*b*-PAMPTMA(+)$_{20}$-NaDC mixed complexes at *CR* = 1 with the presence of 50 mM NaCl. The average width (*W*) of the bundles was analysed (yellow lines) to be 17.6 ± 3.6 nm, which is smaller than those formed without salt. The copolymer concentration was 0.1 wt%.

### 2.5.5 PNIPAM$_{48}$-*b*-PAMPTMA(+)$_{20}$ block copolymer-NaDC system

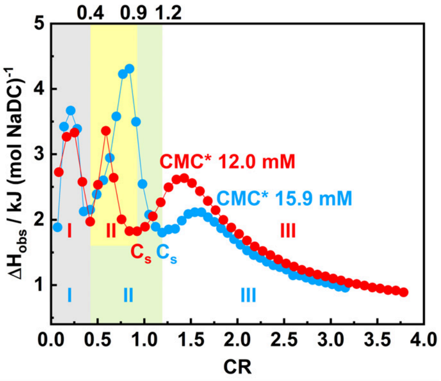


**Figure S27.** ITC curves for the titration of 200 mM NaDC solution into 0.5 wt% aqueous solutions of PNIPAM$_{48}$-*b*-PAMPTMA(+)$_{20}$ (light blue) and PNIPAM$_{65}$-*b*-PAMPTMA(+)$_{20}$ (red), respectively.

## 2.6 Chirality recognition of hexagonally packed supramolecular helices

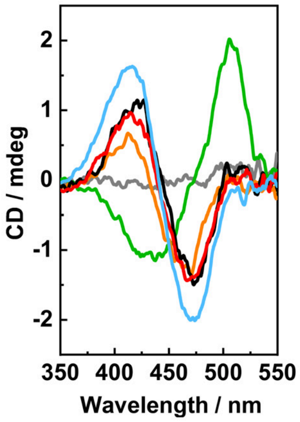


**Figure S28.** CD spectra of 1 mM (green) and 30 mM (orange) NaDC solutions, the PNIPAM$_m$-*b*-PAMPTMA(+)$_{20}$-NaDC mixtures at *CR* = 0.5 (*m* = 65, red, *m* = 48, light blue), the PAMPTMA(+)$_{130}$-NaDC mixture at *CR* = 0.5 (black), and BR in water (gray). The polymer concentration was 0.1 wt%. All solutions contain 100 μM BR.

## Movie Captions

**Movie S1. Cryo-ET reconstruction of the bundles.** Cryo-ET reconstruction (top-view) for the bundles of supramolecular helices formed in the $PNIPAM_{65}$-*b*-PAMPTMA$(+)_{20}$-NaDC mixed complexes at *CR* = 1.

**Movie S2. Cryo-ET reconstruction of the cross section of the bundle.** Cryo-ET reconstruction (cross section) for the bundle marked with 1 in Figure S13. For visual clarity, the movie was created in such a way that each frame represents a rolling average of 50 consecutive cross-sectional positions in the original tomogram, taken along the bundle of supramolecular helices. The yellow label shows relative distances from the arbitrary starting position on the bundle.

**Movie S3. Cryo-ET reconstruction of a section of the bundle.** Isosurface view of a section of the reconstructed bundle from Movie S2.

**Movie S4. Cryo-ET reconstruction of the toroids and bundles.** Cryo-ET reconstruction (top-view) for the bundles and toroids of supramolecular helices formed in the $MPEG_{45}$-*b*-PAMPTMA$(+)_{21}$-NaDC mixed complexes at *CR* = 1.

**Movie S5. Cryo-ET reconstruction of the toroids and bundles.** Cryo-ET reconstruction (top-view) for the same sample as in Movie S4 but from z-slices direction.

**Movie S6. Cryo-ET reconstruction of the entire toroid and bundles.** Cryo-ET reconstruction (overview) for the entire one toroid and two bundles (the same sample as in Movies S4 and S5).

## Reference

[1] S. Bayati, K. Zhu, L. T. T. Trinh, A.-L. Kjøniksen, B. Nyström, *J. Phys. Chem. B* **2012**, *116*, 11386-11395.
[2] R. Pamies, K. Zhu, A.-L. Kjøniksen, B. Nyström, *Polym. Bull.* **2009**, *62*, 487-502.
[3] M. A. Behrens, M. Lopez, A.-L. Kjøniksen, K. Zhu, B. Nyström, J. S. Pedersen, *Langmuir* **2012**, *28*, 1105-1114.
[4] D. N. Mastronarde, *J. Struct. Biol.* **2005**, *152*, 36-51.
[5] C. A. Schneider, W. S. Rasband, K. W. Eliceiri, *Nat. Methods* **2012**, *9*, 671-675.
[6] J. R. Kremer, D. N. Mastronarde, J. R. McIntosh, *J. Struct. Biol.* **1996**, *116*, 71-76.
[7] E. F. Pettersen, T. D. Goddard, C. C. Huang, G. S. Couch, D. M. Greenblatt, E. C. Meng, T. E. Ferrin, *J. Comput. Chem.* **2004**, *25*, 1605-1612.
[8] J. Janiak, S. Bayati, L. Galantini, N. V. Pavel, K. Schillén, *Langmuir* **2012**, *28*, 16536-16546.
[9] a) A. A. D'Archivio, L. Galantini, E. Giglio, A. Jover, *Langmuir* **1998**, *14*, 4776-4781; b) A. Jover, F. Fraga, F. Meijide, J. V. Tato, J. Cautela, A. Del Giudice, M. C. di Gregorio, *J. Colloid Interface Sci.* **2021**, *604*, 415-428; c) F. Lazzari, B. D. Alexander, R. M. Dalgliesh, J. Alongi, E. Ranucci, P. Ferruti, P. C. Griffiths, *Polymers* **2020**, *12*, 900.
[10] a) G. Conte, R. Di Blasi, E. Giglio, A. Parretta, N. Pavel, *J. Phys. Chem.* **1984**, *88*, 5720-5724; b) A. Rich, D. M. Blow, *Nature* **1958**, *182*, 423-426.
[11] a) N. V. Hud, K. H. Downing, *Proc. Natl. Acad. Sci.* **2001**, *98*, 14925-14930; b) N. V. Hud, I. D. Vilfan, *Annu. Rev. Biophys. Biomol. Struct.* **2005**, *34*, 295-318.
[12] M. Thongngam, D. J. McClements, *J. Agric. Food Chem.* **2004**, *52*, 987-991.
[13] G. Olofsson, W. Loh, *J. Braz. Chem. Soc.* **2009**, *20*, 577-593.
[14] a) B. R. Simonović, M. Momirović, *Microchim. Acta* **1997**, *127*, 101-104; b) D. Madenci, S. Egelhaaf, *Curr. Opin. Colloid Interface Sci.* **2010**, *15*, 109-115; c) R. Ninomiya, K. Matsuoka, Y. Moroi, *Biochim. Biophys. Acta* **2003**, *1634*, 116-125; d) P. Garidel, A. Hildebrand, R. Neubert, A. Blume, *Langmuir* **2000**, *16*, 5267-5275; e) I. J. Arroyo-Maya, D. J. McClements, *Biochim. Biophys. Acta* **2016**, *1860*, 1026-1035.
[15] C. Lopez-Pena, I. J. Arroyo-Maya, D. J. McClements, *Food Hydrocolloids* **2019**, *87*, 352-359.
[16] G. BAI, P. LOU, Y. WANG, C. FAN, Y. WANG, *SCIENTIA SINICA Chimica* **2014**, *44*, 1041-1049.
[17] A. Maestre, P. Guardado, M. L. Moyá, *J. Chem. Eng. Data* **2014**, *59*, 433-438.
[18] S. Paula, W. Sues, J. Tuchtenhagen, A. Blume, *J. Phys. Chem.* **1995**, *99*, 11742-11751.
[19] K. Matsuoka, Y. Moroi, *Biochim. Biophys. Acta* **2002**, *1580*, 189-199.
[20] A. Hildebrand, P. Garidel, R. Neubert, A. Blume, *Langmuir* **2004**, *20*, 320-328.
[21] A. Roda, A. F. Hofmann, K. J. Mysels, *J. Biol. Chem.* **1983**, *258*, 6362-6370.
[22] a) K. C. Tam, E. Wyn-Jones, *Chem. Soc. Rev.* **2006**, *35*, 693; b) M. Bonnaud, J. Weiss, D. J. McClements, *J. Agric. Food Chem.* **2010**, *58*, 9770-9777; c) W. Loh, C. Brinatti, K. C. Tam, *Biochim. Biophys. Acta* **2016**, *1860*, 999-1016.
[23] B. Peng, X. Han, H. Liu, K. C. Tam, *J. Colloid Interface Sci.* **2013**, *412*, 17-23.
[24] M. Uchman, M. Gradzielski, B. Angelov, Z. Tošner, J. Oh, T. Chang, M. Štěpánek, K. Procházka, *Macromolecules* **2013**, *46*, 2172-2181.
[25] H. Amenitsch, H. Edlund, A. Khan, E. F. Marques, C. La Mesa, *Colloids and Surfaces A: Physicochem. Eng. Aspects* **2003**, *213*, 79-92.

## Author Contributions

**Guanqun Du**
Formal analysis: Equal
Funding acquisition: Equal
Investigation: Lead
Methodology: Equal
Validation: Equal
Visualization: Lead
Writing – original draft: Lead
Writing – review & editing: Lead

**Domagoj Belić**
Formal analysis: Supporting
Investigation: Equal
Methodology: Equal
Validation: Equal
Writing – review & editing: Equal

**Alessandra Del Giudice**
Investigation: Equal
Resources: Equal
Validation: Equal
Writing – review & editing: Equal

**Viveka Alfredsson**
Funding acquisition: Equal
Methodology: Equal
Resources: Equal
Supervision: Supporting
Writing – review & editing: Equal

**Anna M. Carnerup**
Methodology: Supporting
Resources: Equal
Validation: Equal
Writing – review & editing: Equal

**Kaizheng Zhu**
Resources: Equal
Validation: Equal
Other: Polymer synthesis: Lead

**Bo Nyström**
Resources: Equal
Validation: Equal
Writing – review & editing: Equal

**Yilin Wang**
Funding acquisition: Equal
Methodology: Equal
Resources: Equal
Supervision: Supporting
Validation: Equal
Writing – review & editing: Equal

# SUPPORTING INFORMATION

**Luciano Galantini**
Conceptualization: Equal
Formal analysis: Equal
Funding acquisition: Equal
Methodology: Equal
Resources: Equal
Supervision: Lead
Validation: Equal
Writing – original draft: Lead
Writing – review & editing: Lead

**Karin Schillén**
Conceptualization: Equal
Formal analysis: Equal
Funding acquisition: Lead
Methodology: Equal
Project administration: Lead
Resources: Lead
Supervision: Lead
Validation: Equal
Writing – original draft: Lead
Writing – review & editing: Lead
